# Resisting Aridification: Adaptation of Sap Conduction Performance in Moroccan Wild Olive Subspecies Distributed Over an Aridity Gradient

**DOI:** 10.3389/fpls.2021.663721

**Published:** 2021-07-02

**Authors:** Jalal Kassout, Mohammed Ater, Sarah Ivorra, Hicham Barbara, Bertrand Limier, Jérôme Ros, Vincent Girard, Laure Paradis, Jean-Frédéric Terral

**Affiliations:** ^1^Laboratoire Botanique Appliquée, Equipe bio-Agrodiversité, Faculté des Sciences, Université Abdelmalek Essaâdi, Tétouan, Morocco; ^2^ISEM, Université de Montpellier, Equipe DBA, CNRS, IRD, EPHE, Montpellier, France; ^3^Associated International Laboratory/International Research Project EVOLEA, INEE-CNRS, France – Morocco, Montpellier, France; ^4^INRAE, Centre Occitanie-Montpellier, Montpellier, France

**Keywords:** drought stress, eco-anatomical analysis, Morocco, hydraulic plasticity, wild olives, wood traits

## Abstract

In the current context of global change, the increasing frequency and the length of drought periods are testing the resistance capacities of plants of dry habitats. However, although the adaptation of plants to drought has been widely studied, the anatomical features of wood influencing the functional responses of plants to drought are still lacking at the intraspecific level, especially for species with a wide geographical distribution. As a result, we have studied the variation of wood anatomical traits related to sap conduction (i.e., vessel surface area, vessel density, and number of vessels joined by radial file) in two wild olive subspecies distributed in Morocco (i.e., *Olea europaea* subsp. *europaea*. var. *sylvestris* and *Olea europaea* subsp. *maroccana*), in relation to various drought conditions. This functional study, based on wood trait measurements of 351 samples from 130 trees and 13 populations, explores potential sap conduction in relation to environmental parameters and as a result, strategies to resist water stress. We found that (1) branch diameter (BD) captured 78% of total wood trait variation, (2) vessel size (SVS) expressed 32% of intraspecific variation according to cambium age, and (3) the positive relationship between SVS and BD could be explained by climate type, vegetation cover changes, and therefore available water resources. Taking into consideration the diameter of the branch as the main factor of anatomical variation, established reaction norms (linear models) at the intrapopulation scale of vessel lumen area according to aridity show for the first time how the functioning of the cambium modulates and controls sap conduction, according to aridity and thus available water resources. They pinpoint the risks incurred by the wild olive tree in the perspective of a dramatic increase in aridity, in particular, the inability of the cambium to produce large enough vessels to efficiently transport sap and irrigate the leaves. Finally, this study opens new and interesting avenues for studying at a Mediterranean scale, the resistance and the vulnerability of wild forms and cultivated varieties of olive to heterogeneous and changing environmental conditions.

## Introduction

The Mediterranean climate is characterized by a seasonal variation between hot, dry summers, and mild, rainy winters. This type of climate induces severe and contrasted stresses to both habitats and species. Drought stress appears to be the most critical and limiting factor affecting plant growth, development, and survival (Choat et al., 2018) notably for several groups of Mediterranean plant species such as *Olea europaea* L. subsp. *europaea*. var. *sylvestris* and *Quercus ilex* L. (Quero et al., 2011). The incidence of drought in the Mediterranean region, with extreme warming and drying trends than in other climatic ecoregions, is imposed by higher temperatures combined with changes in precipitation patterns (Cramer et al., 2018). Furthermore, changes in vegetation cover (VC) and land use modify the balance in surface energy, affect surface temperature and soil water availability, and consequently lead to increasing rates of aridification (Pielke et al., 2011). The landscape aridification caused by an increase in temperatures, in the frequency of drought periods, and in human pressures is one of the most obvious consequences of global change (Parmesan and Yohe, 2003). Therefore, the predicted increase in frequency and severity of drought are expected to have harmful effects on trees and forest ecosystems (Allen et al., 2010). Importantly, the tolerance of drought stress under conditions of increasing aridity determines the survival and distribution of plant species (Allen et al., 2010), and the increase in the duration and severity of annual drought is now endangering even the most drought-tolerant species (Choat et al., 2012).

Water stress in trees is associated with scarcity of rainfall or elevated potential evapotranspiration under high temperatures. The impact is particularly high on “rear-edge” populations located at low altitudes, where drought can be extreme (Hampe and Petit, 2005). In trees, this may have two physiological consequences, namely, hydraulic failure and carbon starvation (Anderegg et al., 2012). Relevantly, water transport through the root–xylem–leaf continuum, considered as the most vital function (Steppe et al., 2015), is mainly controlled by a combination of stomatal regulation and xylem characteristics (Choat et al., 2012). Under drought conditions and low water availability, plants restrict water loss through stomatal closure, which reduces leaf transpiration and prevents hydraulic failure (Perez-Martin et al., 2014). Hydraulic failure results from cavitation, which is associated with prolonged and severe drought and leads to tree mortality (Choat et al., 2018). In addition, efficient hydraulic conductivity in the xylem, hydraulic architecture, and growth potential are all interrelated with variations in hydraulic traits critically affecting plant responses to drought (Choat et al., 2018). Resistance to cavitation constitutes an important filter for adaptation to drought both at the interspecific (Maherali et al., 2004) and intraspecific (Lamy et al., 2011) levels. This, together with the ability of species to adjust their hydraulic systems, determines developmental success and competitiveness in contrasted environments (Fonti et al., 2009). As a result, variation in hydraulic traits along drought gradients or within ecosystems can be used to separate adaptation to contrasted levels of water availability (Brodribb et al., 2020). In the context of climate change, the use of such “mechanistic” approaches can reliably be used to explain and predict the broad patterns of plant adaptive strategies to drought (Volaire, 2018).

In general, tolerance to cavitation has mainly been studied by ecophysiological approaches. However, a number of studies have highlighted the contribution of traits associated with wood anatomy to the maintenance of hydraulic efficiency and ecophysiological processes, particularly under drought conditions (Anderegg et al., 2016). Wood density (number of vessels/wood surface unit) seems to be a good predictor of vulnerability and subsequent resistance to cavitation at both the inter- and intra-specific levels (Rosner et al., 2007). An increase in vessel density, associated with a concomitant decrease in diameter or surface area (commonly expressed as the mean vessel lumen area) has been shown to provide an efficient adjustment to the hydraulic systems of olive trees for coping with water stress (Terral et al., 2004). These adjustments relate to a major trade-off between security/efficiency and safety of conduction (Terral et al., 2004).

**Figure 1:**
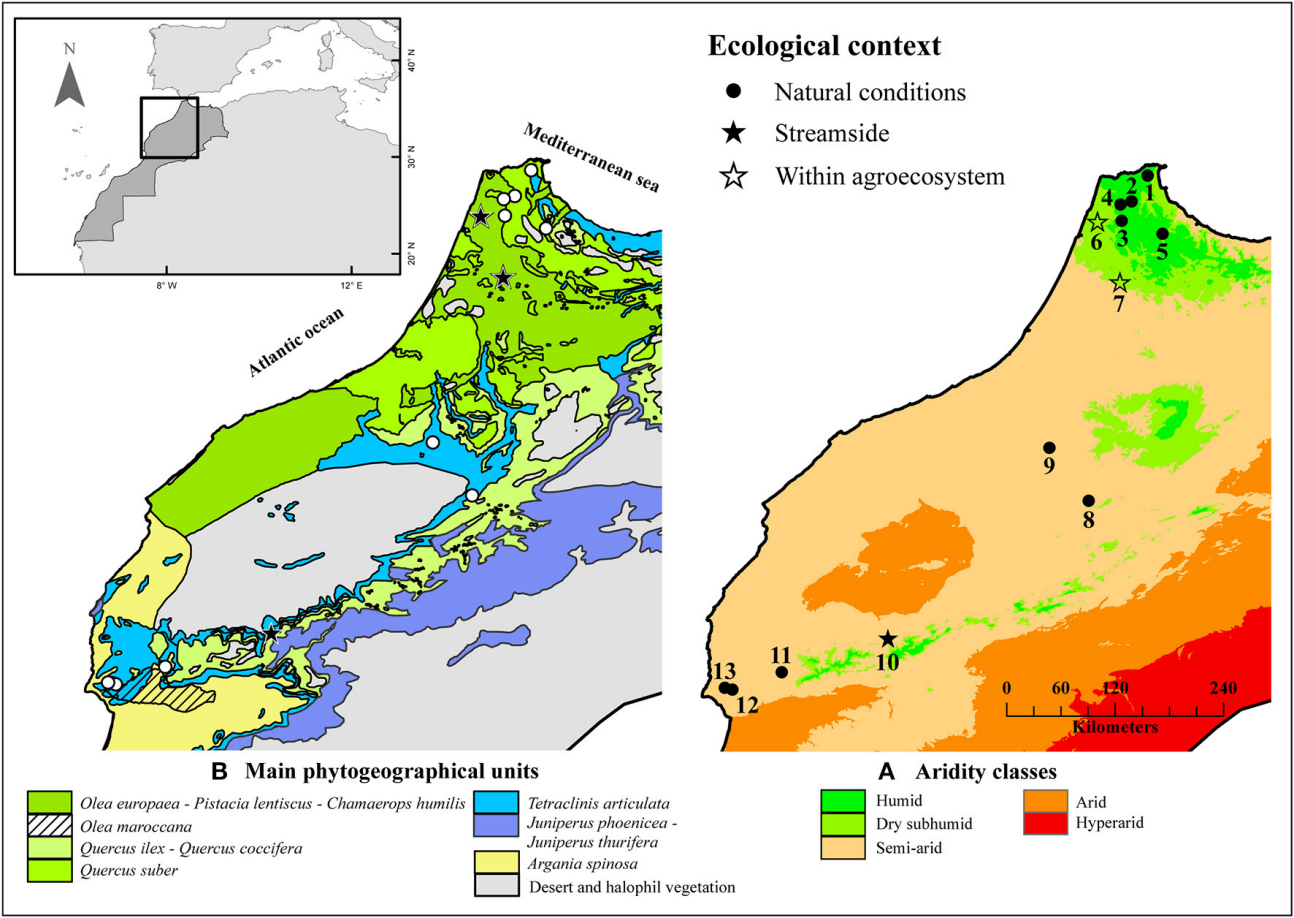
Biogeographical and bioclimatic context of *Olea europaea* L. populations (wild olive or oleaster and Moroccan olive) from which the wood samples were collected and analyzed (Kassout et al., 2019). **(A)** Aridity classes and **(B)** phytogeographical units of the studied area.

In the Mediterranean biogeographical region, scrub is one of the most important and widespread vegetation types. Evergreen shrubs dominate such vegetation, usually with small sclerophyllous leaves, as part of their drought tolerance strategy (Lo Gullo and Salleo, 1988). Thus, some Mediterranean genera exhibit a wide range of mechanisms to withstand drought, and this implies that several morphophysiological and biochemical traits are involved in the control of water loss (West et al., 2012; Kassout et al., 2019). Olive (*O. europaea* L.) is the most iconic sclerophyllous Mediterranean tree. It is represented by two subspecies in Morocco. The first, wild olive or oleaster, *O. europaea* subsp. *europaea* var. *sylvestris*, is the ancestor of the whole cultivated varieties grouped under the *O. europaea* subsp. *europaea* var. *sativa* denomination. The second is the Moroccan olive, *O. europaea* subsp. *maroccana*, endemic to the south-eastern part of the country (Médail et al., 2001). Wild olive or oleaster is very widespread around the Mediterrannean around the Mediterranean Basin where it is a fundamental structuring element of many woody plant communities (Gianguzzi and Bazan, 2019). In Northern Morocco, oleaster is the keystone species of traditional agro-ecosystems in which it is preserved and used for rootstock, oil, shade, and fodder, and for religious reasons (Aumeeruddy-Thomas et al., 2014). However, its geographical distribution is highly fragmented mainly due to anthropogenic disturbances to natural habitats but amplified by climatic changes and aridification (Gianguzzi and Bazan, 2019). In southern areas, oleaster may be found in sympatry with the endemic Moroccan olive, *O. europaea* subsp. *maroccana*, and associated with other emblematic species such as the Argan tree (*Argania spinosa*, Sapotaceae) and Arar tree (*Tetraclinis articulata*, Cupressaceae; Médail et al., 2001; Figure 1).

In a Mediterranean bioclimatic and biogeographical context, drought tolerance is a key component of adaptive responses of trees to aridification, particularly in “rear edge” populations. In Morocco, from North to South, the wild olive tree is widely distributed in a range of plant communities along a latitudinal gradient of aridity. A recent study demonstrates the existence of functional responses to aridity, which is mainly reflected by a “trade-off” between leaf functional traits (Kassout et al., 2019). In some ways, this ecogeographical gradient of aridity indicates future climate changes and upcoming pressures on tree populations. However, anatomical variations relating to drought in wild olive have been less studied (Lo Gullo and Salleo, 1988; Terral, 2000; Terral et al., 2004) with published data concentrated on cultivated olive trees (Trifilò et al., 2007; Guerfel et al., 2009; Ennajeh et al., 2010).

This study explores the ecological plasticity of two wild olive subspecies (Oleaster: *O. europaea* subsp. *europaea* var. *sylvestris* and Moroccan wild olive: *O. europaea* subsp. *maroccana*) in relation to variation and covariation of wood anatomical traits associated with sap conduction. It aims to bring new insights into the functioning of the tree, to understand how sap conduction is optimized, and an increasing aridification of environmental conditions can be resisted despite a decrease in available water resources. In particular, we analyzed wood anatomical traits along a gradient of increasing aridity to address the following questions: (i) do wild olive populations differ in their responses to drought and aridification? and (ii) is this variability influenced by other factors such as tree age and local environmental conditions?

## Materials and Methods

### Sampling Procedure and Wood Material

A total of 13 populations and several trees per population (Supplementary Figure 1) were sampled to include a wide range of variation in ecological conditions and tree age (Figure 1, Tables 1, 2). Plant materials (351 samples) consisted of wood samples collected from trees (130 trees) of wild olive or oleaster (WO) and Moroccan olive (MO). The wood samples were collected at different heights of the trees in the form of branch sections varying in diameter (and age) ranging from 2.2 to 19.4 mm (Supplementary Table 5). The investigated populations cover the main ecological situations with which wild olives are associated in Morocco (e.g., natural conditions within forest or matorral vegetation types, streamside populations, and within traditional agroecosystems; Tables 1, 2). Only section samples with the medulla in the center of the branch were retained to avoid any impact of asymmetrical biomechanical constraints on the size of wood characters. Following the Braun-Blanquet method (1964) and using a quadrat with an area of 200 m^2^ for each population, the vegetation cover was estimated distinguishing both trees and shrub layers (Table 2). The herbaceous layer was not taken into consideration because the surveys were conducted over 2 years (2016 and 2017) and never at the same time. Moreover, restricting the sampling to woody species was useful due to similarities in growth forms between trees and shrubs. Anthropogenic disturbance such as fire frequency, grazing, wood cutting, and grafting on WO trees was also recorded (Table 2).

**Table 1:** Geographical coordinates and mean climatic data of the sampled sites listed according to their subspecies affiliation and from north to south.

**Site names**	**Olive subspecies**	**Latitude (^**°**^)**	**Longitude (^**°**^)**	**Altitude (m)**	**MAT (^**°**^C)^c^**	**TMA (^**°**^C)^c^**	**TMI (^**°**^C)^c^**	**MAP (mm/year)^c^**	**AI**
1. Tlat Taghramt	WO	35.7889	−5.4681	293	16.9	29.5	6.3	801	0.708
2. Bni Harchim	WO	35.5510	−5.6198	150	18.0	30.4	7.3	779	0.652
3. Bni Arous	WO	35.3567	−5.7185	90	18.1	31.3	6.5	781	0.621
4. Dar Chaoui	WO	35.5205	−5.7295	64	18.1	30.0	7.7	751	0.626
5. Dar Akouba	WO	35.2305	−5.3095	322	17.7	32.3	5.6	774	0.611
6. Tnin Sidi Yemeni^a^	WO	35.3526	−5.9580	126	17.7	30.4	6.1	765	0.609
7. Mesmouda^a^	WO	34.7499	−5.7341	196	18.0	33.9	5.0	805	0.586
8. El Ksiba	WO	32.5745	−6.0442	900	16.6	36.7	1.4	721	0.465
9. Moulay Bouazza	WO	33.1021	−6.4357	745	16.6	35.3	2.2	545	0.369
10. Asni^b^	WO	31.2076	−8.0413	953	15.6	33.2	0.2	420	0.284
11. Argana	MO	30.8716	−9.0973	1010	16.4	27.3	4.5	296	0.240
12. Immouzzer	MO	30.6973	−9.5891	595	16.0	25.5	5.2	307	0.268
13. Issi-Adgil	MO	30.7161	−9.6649	353	16.7	25.1	6.4	286	0.253

*Climatic variables were extracted from Worldclim database (Fick and Hijmans, 2017) and the CGIAR Global Aridity and PET Database (Zomer et al., 2007), and abbreviated as follows: MAT, mean annual temperature (°C); MAWM, mean annual temperature of the warmest month (°C); MACM, mean annual temperature of the coldest month (°C); MAP, mean annual precipitation (mm); AI, aridity index (unit less, calculated as PET/MAP, PET: potential evapotranspiration in mm/year) (UNEP, 1997)*.

a *Populations within agroecosystems*,

b *streamside population, and*

c *33 years-average values were used for climatic variables. High AI values refer to humid conditions and low values refer to arid conditions. WO, wild olive or oleaster (Olea europaea subsp. europaea var. sylvestris); MO, Moroccan olive (Olea europaea subsp. maroccana)*.

**Table 2:** Number of samples analyzed and main ecological features per site.

**Site names**	**ID Pop**	**Samples number**	**Bioclimate**	**Vegetation series (types)**	**VC%**		**Human disturbances**			
					**Trees**	**Shrubs**	**GR**	**WC**	**GA**	**FI**
1. Tlat Taghramt	TLT	28 [**10**]^c^	Subhumid/humid	Kermes oak matorral (*Quercus coccifera*)	50	50	0	2	2	1
2. Bni Harchim	BNH	24(3)^d^ [**10**]	Subhumid/humid	Matorral dominated by wild olive (*O. e*. subsp. e. var. *sylvestris*), pistachio mastic tree (*Pistacia* textitlentiscus) and Mediterranean dwarf palm (*Chamaerops humilis)*	10	50	0	2	3	0
3. Bni Arous	BNA	26 [**10**]	Dry subhumid		10	60	0	2	2	1
4. Dar Chaoui	DAC	29 [**10**]	Dry subhumid		20	90	0	2	2	1
5. Dar Akouba	DAR	19 [**8**]	Dry subhumid		15	60	1	3	2	1
6. Tni Sidi Yemeni^a^	TNY	31 [**10**]	Dry subhumid		40	20	0	1	3	0
7. Mesmouda^a^	MES	31 [**10**]	Dry subhumid		60	30	1	2	2	1
8. El Ksiba	KSB	26 [**11**]	Semiarid	Matorral with wild olive (*O. e*. subsp. *e*. var. *sylvestris*), barbary thuya (*Tetraclinis articulata)* and pistachio mastic tree (*Pistacia lentiscus*)	20	80	0	1	2	0
9. Moulay Bouazza	MBO	21 [**8**]	Semiarid	Barbary thuya *(Tetraclinis articulata)* woodlands	20	5	0	1	3	0
10. Asni^b^	ASN	29 [**10**]	Semiarid	Barbary thuya *(Tertraclinis articulata)* and Phoenician juniper (*Juniperus phoenicea*) woodlands	20	70	0	2	3	0
11. Argana	ARG	33(11)^d^ [**15**]	Semiarid	Barbary thuya (*Tetraclinis articulata*), Argan	10	30	0	2	3	0
12. Immouzzer	IMO	25 [**9**]	Semiarid	tree (*Argania spinosa)* and	20	30	0	2	3	0
13. Issi-Adgil	ISS	15 [**8**]	Semiarid	Moroccan wild olive (*O. e*. subsp. *maroccana)* woodlands	40	80	0	1	2	0

*Vegetation series (types) relates to Benabid and Fennane (1994). VC%, vegetation cover for tree and shrub layers, estimated by following the Braun-Blanquet cover-abundance scale method (1964). Human disturbance was qualitatively estimated on four scales: 0: absent, 1: low, 2: medium, and 3: intense. GR, grafting (grafting on O. europaea subsp. europaea var. sylvestris); WC, wood cutting; GA, grazing; FI, fire frequency. The number of sampled sites is identical to Figure 1*.

a *Populations within the traditional agroecosystem area*,

b *streamside population*,

c *bold numbers between [ ] indicate the number of trees sampled in each population, and*

d *the numbers between brackets indicate additional samples from streamside location*.

### Preparation of Samples and Transverse Sections for Microscopy

A sliding microtome (G.S.L.1, Microm International GmbH, Walldorf, Germany) was used to obtain thin transverse sections (8 μm) from wood samples. Histological preparations were then obtained by staining transverse sections with one to one mixture of Fast Green (2%) and Safranin (1%) solutions. Sections were subsequently dehydrated using a series of ethanol solutions (96, 70, and 40%), washed with xylene (Kassout et al., 2016), and embedded in Canada balsam as permanent slides for future observations, measurements, and analysis.

### Measurements of Wood Anatomical Traits

**Figure 2:**
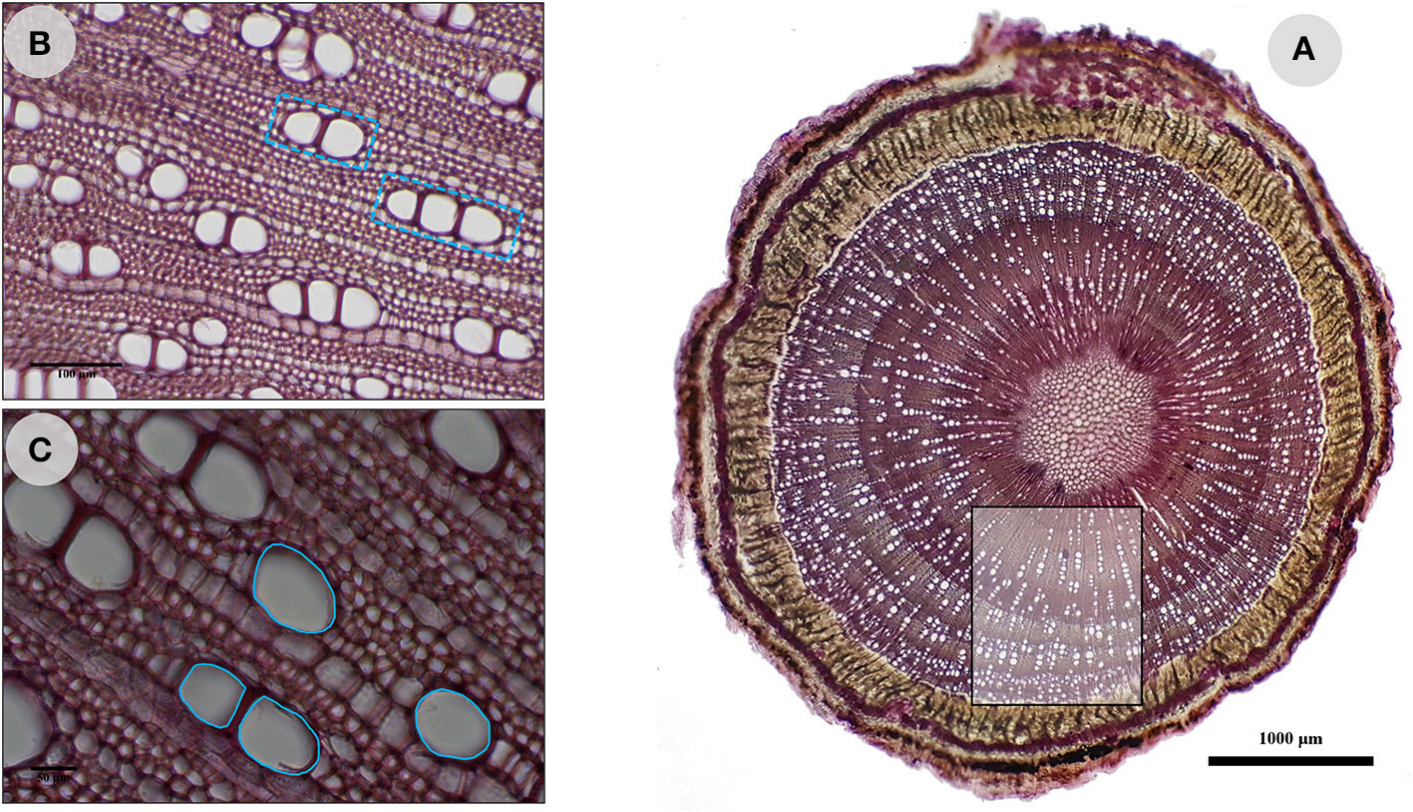
Wood anatomy of *O. europaea* L. subsp. *europaea* var. *sylvestris* and measured anatomical characters. **(A)** Vessel density (DVS, number of vessels/mm^2^); **(B)** number of vessels joined in radial files (NVS, N/group of vessels); **(C)** vessel surface area (SVS, μm^2^). The scales are 100 and 50 μm for **(B,C)**, respectively.

The eco-anatomical approach was used to measure wood traits using a light transmission microscope connected to an image analysis system (Limier et al., 2018). This approach involved measuring anatomical wood characters (or traits) followed by statistical analyses of data. Then, the results were interpreted from taxonomic, functional, biological, and ecological viewpoints (Terral et al., 2004). For each wood sample, whose branch diameter (BD, mm) was previously measured, three anatomical characters involved in sap conduction were measured: vessel density (DVS, number of vessels/mm^2^; Figure 2A), number of vessels joined in radial files (NVS, N/group of vessels; Figure 2B), and vessel surface area (SVS, μm^2^; Figure 2C). The measurements were taken in the youngest (peripheral) part of the wood (from 1 to about 3 years old) under a magnification of 100×, 200×, and 400× for DVS, NVS, and SVS, respectively.

### Reliability and Reproducibility of Measurements

For an optimal evaluation of anatomical traits involved in sap conduction and to obtain reliable mean values, numerous measurements were taken for each anatomical trait from test wood samples (Limier et al., 2018). On test samples, cumulative average curves were established, comparing the cumulative average with the number of measurements taken for each anatomical trait. When the curve stabilizes, the corresponding number of measurements represents a reliable estimate of the trait mean value. To verify the absence or existence of errors that might affect the validity of measurements of wood anatomical traits, several tests were carried out over the course of different days: a sample measured by two operators and a sample measured twice by the same operator. The forward statistical analysis was carried out after having (1) determined the number of measurements required for a reliable assessment of anatomical traits and (2) checked that the measurement errors were negligible using a parametric *t*-test or non-parametric Mann–Whitney *U*-test carried out on data acquired by the same operator and two different operators. Repeatability and reproducibility tests were carried out on a test sample from the “Dar Chaoui” population. Cumulative average curves were established to obtain a reliable estimate of the mean value per anatomical trait to be used.

### Statistical Analysis

In order to assess the variability of wood anatomical traits according to age (assessed by BD), a principal component analysis (PCA) was carried out using the 351 wood samples and 4 quantitative variables, namely the 3 anatomical traits relating to sap conduction (DVS, NVS, and SVS) and the BD. Then, a variance decomposition procedure was used (Albert et al., 2010) to quantify trait variance in relation to branch age across different scales and ecological situations (i.e., sample, population, aridity, and VC). The “lme” function in the “nlme” package (Pinheiro et al., 2019) was used to fit a general linear model using the restricted maximum likelihood method (RMEL) across the studied levels, and then the “varcomp” function in the “ape” package (Paradis et al., 2004) was used to extract variance expressed at each level. Once the impact of branch age on anatomical traits is demonstrated and the anatomical traits more significantly linked to BD is determined (Spearman's correlation analysis), two different statistical approaches were performed (with results compared).

First, we carried out linear regression analyses, population by population. Normality of data was tested using the Shapiro–Wilk normality test and non-normal distributed data are log-transformed. Finally, parameters of the linear models (*y*-intercept defining the elevation of the line and slope) were compared with each other, through integration with the aridity index (AI, after UNEP, 1997), the growth ecological conditions (i.e., natural, streamside, or agroecosystem), the vegetation context, and the VC.

The second approach consisted in a standardized major axis (SMA) regression (Warton et al., 2012), also known as reduced major axis slopes. For 246 samples from wild olive trees growing in natural conditions (Table 1), the SMA regression was used to analyze the relationships between anatomical traits and BD by assessing the effect of aridity and VC on this potential interrelation. Three different classes based on the AI (UNEP, 1997) were first compared (i.e., Ca1: subhumid/humid, Ca2: dry subhumid, and Ca3: semiarid). In the second stage, the VC (only shrubs) was integrated, allowing the testing of seven distinct classes [Cavc1: subhumid/humid + medium VC (+33–66%), Cavc2: subhumid/humid + high VC (+66–100%), Cavc3: dry subhumid + high VC, Cavc4: dry subhumid + medium VC, Cavc5: semiarid + high VC, Cavc6: semiarid + medium VC, and Cavc7: semiarid + low VC (0–33%)]. This approach allowed the description of the best-fit scaling relationship between the studied anatomical traits on log–log axes, allowing scaling relationships among variables to be compared across different groups. The analysis was carried out using the R software (R 3.3.3; R Development Core Team, 2015). The *smatr* package version 3 was used (Warton et al., 2012) for the SMA regressions. Finally, to explore specific anatomical strategies of wild olive trees in a heterogeneous environment (i.e., aridity, plant community types, and VC), we have investigated intrapopulation correlations among wood anatomical traits.

## Results

### Reliability, Repeatability, and Reproducibility of Measurements

The number of measurements required for an optimal evaluation proved to be at least 50 for both SVS and NVS and 25–30 for DVS (Supplementary Figure 2). The frequency distributions of SVS and DVS calculated using the Shapiro–Wilk test follow a normal distribution (Supplementary Figure 3, Supplementary Tables 1, 2). Based on these results, mean comparisons were made using the parametric *t*-test. The results showed that eco-anatomical data acquired by the same operator during two distinct sessions or by two different operators do not differ significantly. In the case of NVS, that is not normally distributed, a non-parametric Mann–Whitney *U*-test identified that the different data sets are comparable (Supplementary Tables 3, 4). Thus, the measurements of the three anatomical characters were repeatable, and the eco-anatomical analysis was reproducible using these anatomical traits.

### Variation of Wood Anatomical Traits

**Figure 3:**
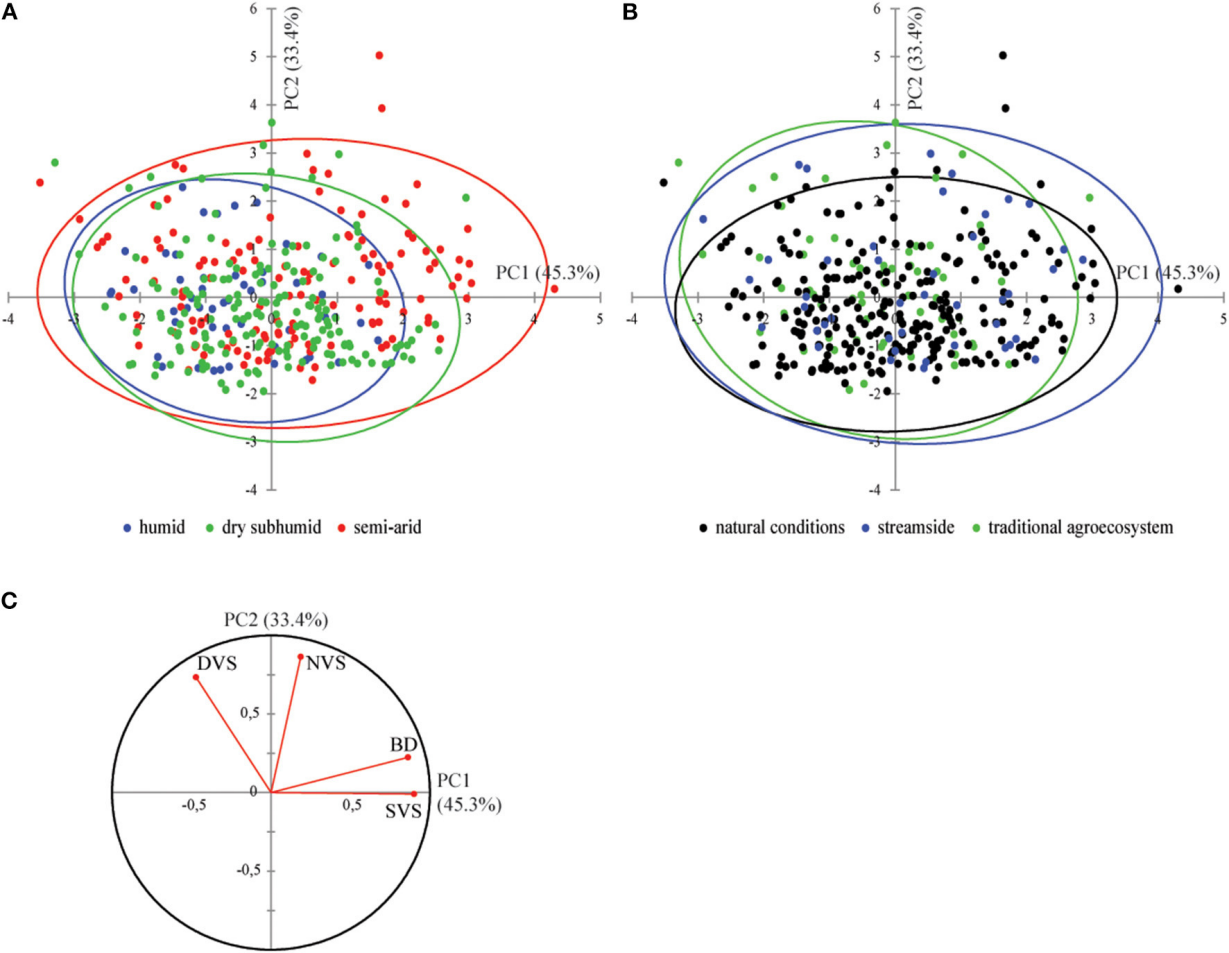
Principal component analysis biplot of axes 1 and 2 (78.7% of variability) of wood olive samples belonging to different categories [**(A)** bioclimatic conditions and **(B)** growing conditions]. Correlations among anatomical traits [**(C)** correlation circle] and contribution of variables in the definition of PCA axes **(C)** are also presented.

The PCA carried out on the quantitative data from the eco-anatomical analysis (351 samples from 13 populations) showed the prevalence of BD in the overall anatomical variability with 78.7% of variance explained by the first two principal axes (Figure 3). Distribution of individuals along axis 1 (45.3%) is mainly explained by differences in BD (Figures 3A,B), with SVS significantly correlated with BD (*R*-Spearman = 0.66, *p* < 0.0001) (Figure 3C). As a result, although they were also related to BD, NVS, and DVS explained the shifting of individuals along axis 2 of the PCA (33.4% of variance) (Figures 3A,B). Furthermore, the variance decomposition of SVS showed that >32% of variance was due to differences in the BD, with <3% due to differences between populations, aridity, or vegetation classes (Supplementary Table 6). The eco-anatomical data have been summarized in Supplementary Table 5.

### Driving Factors of Wood Anatomical Trait Variability

**Figure 4:**
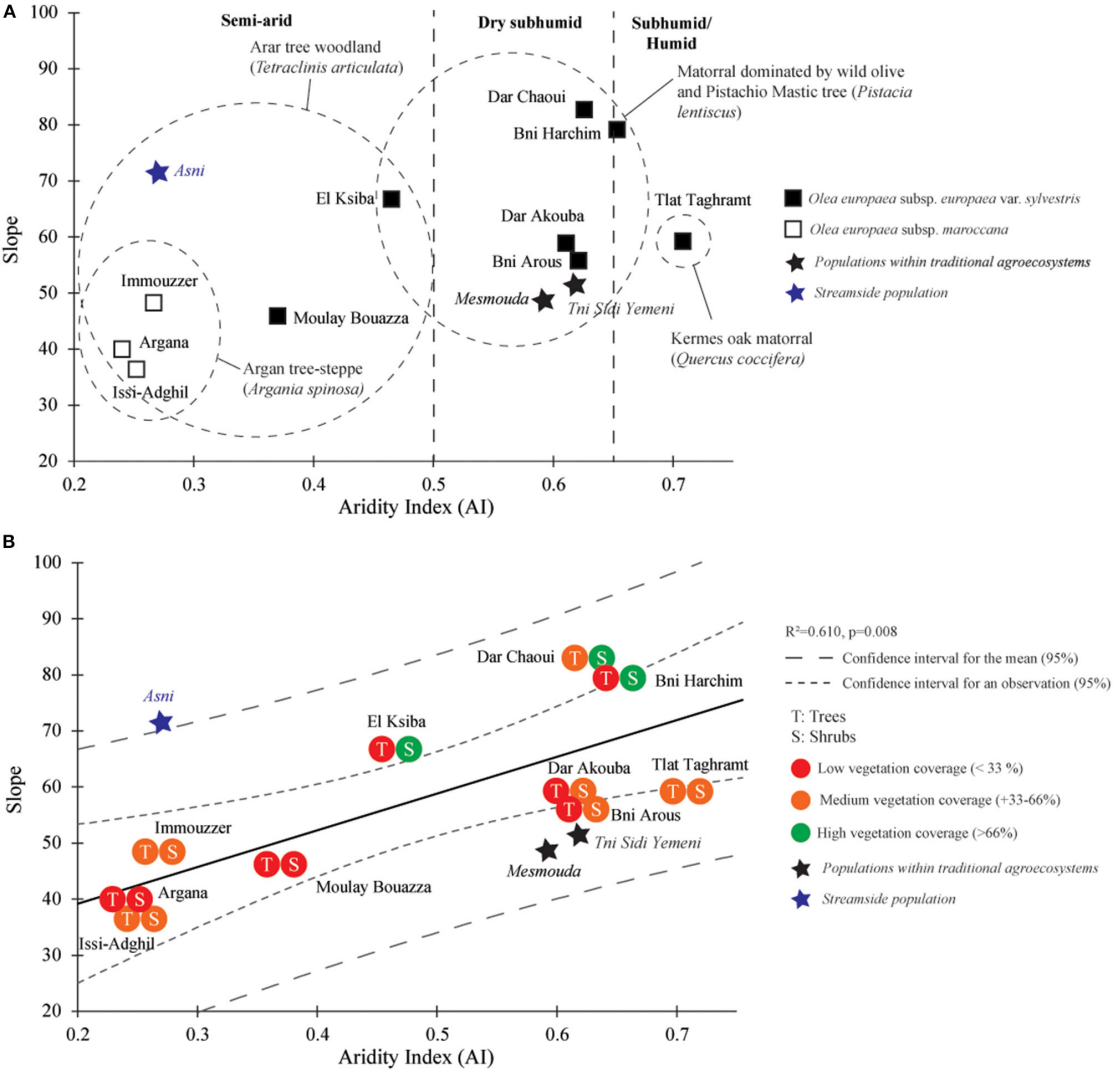
**(A)** Biplot separating contrasted populations using the aridity index (AI) and slope of fitted lines from regression analysis relating vessel surface area (SVS) and branch diameter (BD). **(B)** Linear regression model of variation in slope of fitted lines from regression analysis relating SVS and BD.

Generally, populations from semiarid bioclimatic conditions were characterized by a fitted line with a low slope, except for “Asni” where trees grow at the edge of intermittent streams (wadi) (Figure 4A). The simple linear regression analysis carried out for each population based on the results evidenced by the PCA showed significant relationships between SVS and BD (Table 3), and notable differences in *y*-intercept and slope of the fitted line among populations. As *y*-intercept and slope were strongly negatively correlated (*R*^2^ = 0.68, *p* = 0.001), only the slope data have been presented. *O. europaea* subsp. *maroccana* populations from *A. spinosa* woodlands exhibited the lowest values of slope (Figure 4A). For the exceptional “Asni” population, the slope appeared comparable with those defining populations from subhumid and humid bioclimatic conditions and characterized by a matorral-type vegetation such as “Bni Harchin” and “Dar Chaoui” (Figure 4A). In contrast to semiarid populations from *A. spinosa* and/or *T. articulata* woodlands, dry subhumid and subhumid/humid populations were identified by higher slope values. Nevertheless, the “Mesmouda” and “Tni Sidi Yemeni,” both situated within traditional agroecosystems, appeared to be anomalous populations as their slope values are similar to semiarid ones (Figure 4A). As an overall trend in the variation of fitted-line slopes in relation to bioclimatic conditions was apparent, the relationship slope = *f* (aridity index, AI) was subsequently modeled by linear regression. Populations from natural conditions and with recorded VC were only included. Populations from streamside and traditional agroecosystem areas were considered as additional statistical individuals but not used to define the model. In the model, a significant relationship between the slope of the fitted line and AI was identified (Figure 4B), showing that the increase in SVS, a consequence of BD, is linked not only to bioclimatic conditions (AI) but also related to the nature of VC.

**Table 3:** Summary of the regression analysis relating vessel surface area (SVS) and branch diameter (BD) at the intrapopulation level.

**Population**	**Growth conditions**	**Number of samples**	**Shapiro–Wilk normality test**				**Linear regression analysis: SVS** **=** **f(diameter)**		
			**Vessel Surface area (SVS**, **μm**^**2**^**)**		**Branch diameter (cm)**				
			**W**	***P*-value**	**W**	***P*-value**	***R*^2^**	**F**	***P*-value**
1. Tlat Taghramt	Normal conditions	28	0.977	0.774	0.981	0.874	0.26	9.23	0.005
2. Bni Harchim	Normal conditions	24	0.940	0.199	0.970	0.708	0.49	18.98	<0.0001
3. Bni Arous	Normal conditions	26 + 3^a^	0.948	0.209	0.502	0.502	0.57	31.99	<0.0001
4. Dar Chaoui	Normal conditions	29	0.964	0.406	0.939	0.092	0.51	29.83	<0.0001
5. Dar Akouba	Normal conditions	19	0.962	0.584	0.929	0.132	0.60	24.99	<0.0001
6. Tni Sidi Yemeni	Traditional agroecosystem	31^b^	0.968	0.474	0.964	0.379	0.29	7.00	0.004
7. Mesmouda	Traditional agroecosystems	31^b^	0.963	0.354	0.942	0.093	0.46	22.74	<0.0001
8. El Ksiba	Normal conditions	26	0.965	0.504	0.969	0.605	0.50	23.94	<0.0001
9. Moulay Bouazza	Normal conditions	21	0.951	0.354	0.931	0.141	0.54	22.11	<0.0001
10. Asni	Streamside	29^a^	0.962	0.358	0.942	0.111	0.58	36.53	<0.0001
11. Argana	Normal conditions	33 + 11^a^	0.949	0.122	0.943	0.084	0.52	33.73	<0.0001
12. Immouzzer	Normal conditions	25	0.944^c^	0.180	0.926^c^	0.072	0.69	51.64	<0.0001
13. Issi-Adgil	Normal conditions	15	0.884	0.055	0.934	0.316	0.52	14.05	0.002

*The results of the normality tests are given for the SVS and BD*.

a *Individual growing in temporary stream border*.

b *Individuals within traditional agroecosystems*.

c *Log-transformed data*.

The second approach based on the SMA showed that SVS was positively scaled against the BD when considering all the samples from wild olive trees (WO and MO) growing under natural conditions. SMA fitted groups across aridity classes revealed differences in slopes (likelihood ratio statistic = 16.71, df = 2, *p* < 0.05) but did not differ in *y*-intercept (Wald statistic = 3.304, df = 2, *p* > 0.05; Table 4A). SVS and BD relationships across aridity classes were positively correlated for the subhumid/humid (Ca1), dry subhumid (Ca2), and semiarid class (Ca3) (Table 4A). Pair-wise slope comparisons among aridity classes showed that the subhumid/humid (Ca1) samples differ significantly from the dry subhumid (Ca2) and semiarid (Ca3) samples (Supplementary Table 7). With increasing aridity, the slope of SVS–BD linear relationship decreases from subhumid/humid conditions with a slope of 1.186 (95% CIs: 0.91–1.53) to semiarid conditions with a slope of 0.652 (95% CIs: 0.57–0.73; Table 4A). When testing for differences across VC classes, SMAs showed differences in slope (likelihood ratio statistic = 28.01, df = 6, *p* < 0.05) and *y*-intercept (Wald statistic = 23.92, df = 2, *p* < 0.05; Table 4B). SVS and BD relationships appeared to be positive and significant for all the classes [“subhumid/humid + medium VC” (Cavc1), “subhumid/humid + high VC” (Cavc2), “dry subhumid + high VC” (Cavc3), “dry subhumid + medium VC” (Cavc4), “semiarid + high VC” (Cavc5), “semiarid + medium VC” (Cavc6), and “semiarid + low VC” (Cavc7); Table 4B].

**Table 4:** Log–log relationships between SVS and BD for the studied samples grouped according to their climatic context **(A)** and to their climatic and vegetation characteristics **(B)**.

	***N***	***R*^2^**	***P*-value**	**Slope (95% CIs)**	**Common slope**	**Intercept (95% CIs)**	**Common elevation**
**A. Bioclimate**							
Subhumid/humid	52	0.28	<0.001	1.186 (0.91–1.53)	**0.0002**	2.215 (2.14–2.28)	0.191
Dry subhumid	74	0.52	<0.001	0.760 (0.63–0.90)		2.253 (2.18–2.32)	
Semiarid	120	0.46	<0.001	0.652 (0.57–0.73)		2.255 (2.19–2.32)	
Pooled	246	0.44	<0.0001	0.758 (0.68–0.83)		2.241 (2.17–2.30)	
**B. Bioclimate and vegetation characteristics**							
Cavc1	28	0.19	0.01	1.205 (0.83–1.73)	**0.000009**	2.180 (2.09–2.26)	**0.0005**
Cavc2	24	0.45	<0.01	1.160 (0.83–1.61)		2.254 (2.17–2.33)	
Cavc3	29	0.44	<0.01	0.85 (0.61–1.18)		2.276 (2.20–2.35)	
Cavc4	45	0.60	<0.01	0.74 (0.61–0.91)		2.238 (2.16–2.30)	
Cavc5	26	0.57	<0.01	0.74 (0.56–0.98)		2.276 (2.20–2.34)	
Cavc6	40	0.60	<0.01	0.51 (0.42–0.61)		2.306 (2.23–2.37)	
Cavc7	54	0.45	<0.01	0.70 (0.57–0.85)		2.206 (2.13–2.28)	
Pooled	246	0.44	<0.0001	0.758 (0.68–0.83)		2.241 (2.17–2.30)	

*Slopes, intercepts, and 95% CIs are given*.

*The bold values are significant at p <0.05. The abbreviations of Cavc classes are described in “Materials and methods” section*.

Pair-wise slope comparisons among the seven classes showed that the samples from semiarid and medium VC conditions (Cavc6) differ significantly from the samples from subhumid/humid and medium VC (Cavc1) and subhumid/humid and high VC (Cavc2) (Supplementary Table 8). Pair-wise *y*-intercept comparisons show that the samples from semiarid and medium VC conditions (Cavc6) may be clearly distinguished from the samples affiliated to the “dry subhumid and medium VC” class (Cavc4) and to “semiarid and low VC” class (Cavc7) (Supplementary Table 8).

### Intrapopulation Correlations Among Wood Anatomical Traits

At the intrapopulation level, a negative correlation between SVS and DVS was recorded for populations situated in subhumid/humid and dry subhumid conditions, except for “Bni Harchim” and “Dar Chaoui” populations. Only the semiarid “Argana” population, where several trees grew by streamside, displayed such correlation. A significant positive correlation between SVS and NVS seemed to characterize a single population (“Immouzzer”—semiarid conditions). Finally, three populations (El Ksiba, Moulay Bouazza, and Issi-Adghil), under semiarid bioclimatic conditions, displayed a positive and significant correlation between DVS and NVS (Table 5, Supplementary Figure 4).

**Table 5:** Pearson correlation coefficients between anatomical traits and BD at the intrapopulation level for all the studied populations.

**Sites**	**Traits**	**DVS**	**SVS**	**NVS**
1 (*n* = 28)	DVS	**1**		
	SVS	–**0.400**	**1**	
	NVS	0.331	0.030	**1**
	BD	−0.303	**0.512**	0.081
2 (*n* = 24+**3**)	DVS	**1**		
	SVS	−0.279	**1**	
	NVS	0.247	−0.004	**1**
	BD	−0.341	**0.699**	−0.215
3 (*n* = 26)	DVS	**1**		
	SVS	–**0.491**	**1**	
	NVS	0.081	−0.198	**1**
	BD	–**0.694**	**0.756**	−0.282
4 (*n* = 29)	DVS	**1**		
	SVS	−0.139	**1**	
	NVS	0.357	−0.005	**1**
	BD	−0.240	**0.724**	0.121
5 (*n* = 19)	DVS	**1**		
	SVS	–**0.738**	**1**	
	NVS	0.000	0.056	**1**
	BD	–**0.592**	**0.771**	0.223
6^a^ (*n* = 31)	DVS	**1**		
	SVS	–**0.651**	**1**	
	NVS	0.217	0.076	**1**
	BD	−0.206	**0.441**	0.010
7^a^ (*n* = 31)	DVS	**1**		
	SVS	–**0.600**	**1**	
	NVS	0.050	0.203	**1**
	BD	–**0.614**	**0.620**	0.344
8 (*n* = 26)	DVS	**1**		
	SVS	0.017	**1**	
	NVS	**0.605**	0.189	**1**
	BD	0.007	**0.707**	**0.440**
9 (*n* = 21)	DVS	**1**		
	SVS	−0.412	**1**	
	NVS	**0.631**	−0.001	**1**
	BD	**-0.609**	**0.733**	−0.147
10^b^ (*n* = 29)	DVS	**1**		
	SVS	–**0.381**	**1**	
	NVS	**0.454**	0.145	**1**
	BD	−0.221	**0.758**	**0.481**
11 (*n* = 33+**11**)	DVS	**1**		
	SVS	–**0.444**	**1**	
	NVS	0.199	0.208	**1**
	BD	−0.291	**0.605**	**0.364**
12 (*n* = 25)	DVS	**1**		
	SVS	−0.006	**1**	
	NVS	0.255	**0.493**	**1**
	BD	0.146	**0.821**	**0.806**
13 (*n* = 15)	DVS	**1**		
	SVS	−0.122	**1**	
	NVS	**0.683**	0.297	**1**
	BD	0.202	**0.721**	0.452

*Bold values are significant at α = 0.05*.

*For site numbers and characteristics, see Tables 1, 2. The number of samples per site is given in brackets, in which bold numbers are samples in streamside conditions*.

a *Populations within traditional agroecosystems*.

b *Streamside population*.

## Discussion

### Sap Conduction Performance in Wild Olive (*O. europaea* L.) Depends on Age

Overall, our results showed that an increase in vessel lumen area (SVS) was positively correlated with BD, and thus to the age of the branch and therefore to the quantity of vascular cambium. In trees, an increase in the size of the vessel was globally linked to the increasing volume of plant parts to be fed by sap, namely the axial apices and the leaves of the last two or more rarely three growth-units (Choat et al., 2012). In this way, the growth and tree development and consequently the cambial function appeared to lead to an overall increase in the amount of conducting tissue (Steppe et al., 2015; Ros et al., 2021). Moreover, with the increased age of the branch, the vascular cambium seemed generally to provide a wood with a lower DVS and of lesser density, from a mechanical point of view. Nevertheless, the overall relationship and thus the standard trade-off between vessel SVS and DVS was not recorded in all the populations (Anderegg et al., 2015). Most of the populations that did not suffer a high hydric deficit [subhumid/humid and dry subhumid populations (except “Bni Harchim” and “Dar Chaoui”)] as well as samples from the “Argana” population, where some trees grew beside a stream, exhibited a negative correlation between SVS and DVS. For these populations, mainly distributed in Northern Morocco and characterized by a matorral-type vegetation, a compromise between SVS and DVS has allowed the tree to maintain a relative constant sap conductance and thus water supply. An overall increase in the size of vessel related to the age of the vascular cambium has already been recorded in other species with a distinct wood structure, such as Mediterranean evergreen oaks (holm oak, *Quercus ilex*, and cork oak, *Quercus suber—*semiring porous wood) (Voulgaridis, 1990; Leal et al., 2007), grapevine (*Vitis vinifera*—ring porous wood) (Limier et al., 2018), and argan tree (*A. spinosa—*diffuse porous wood) (Ros et al., 2021).

### Aridity Affects Significantly Anatomical Traits in Wild Olive (*O. europaea* L.)

**Figure 5:**
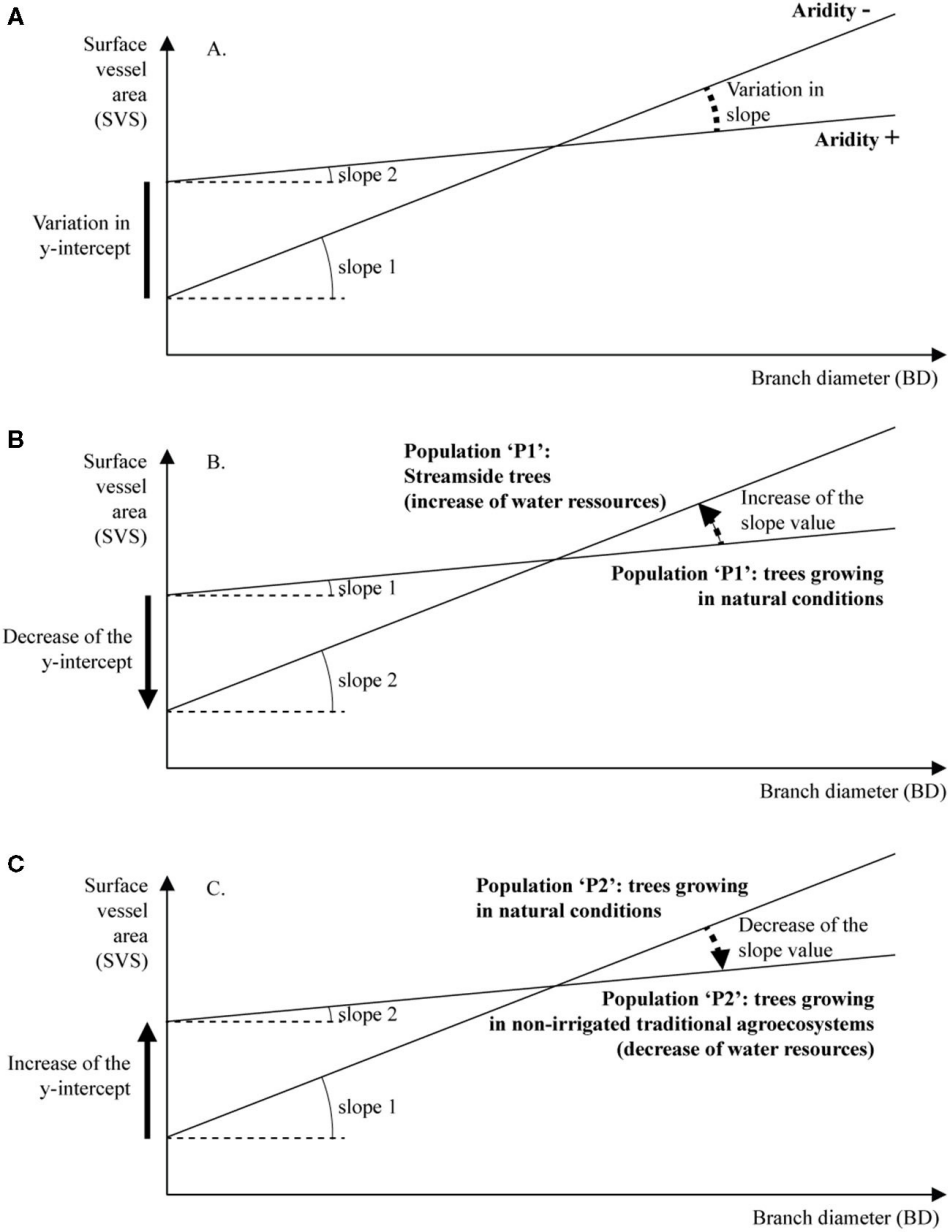
Conceptual summary of variations in slope and intercept of linear models relating SVS and BD, according to water resources [**(A)** in natural conditions, **(B)** in streamside conditions, and **(C)** in non-irrigated cultivation areas].

At the intrapopulation level, variations in SVS in relation to BD modeled independently using the simple regression analysis and a SMA regression approach have shown that the increase in the size of the vessel is modulated in relation to aridity (AI) and other local environmental factors, such as additional water resources provided by temporary streams and the evapotranspirational impact of VC (Figure 5). Previously, without considering the branch age, the authors demonstrated that unfavorable conditions for tree growth could lead to a decrease in the size of vessel and correlatively, an increase in vessel density (Carlquist, 1988). The size of the vessel in evergreen Mediterranean wood trees such as olive and oaks has been reported as strongly influenced by seasonal or annual precipitations (Terral and Arnold-Simard, 1996; Figueiral and Terral, 2002; Terral et al., 2004 for the olive and, Eckstein and Frisse, 1979; Woodcock, 1989 for oaks). The anatomical response was even more important when trees are irrigated, as shown in the study by Terral and Durand (2006) for cultivated olives.

Moreover, previous studies have demonstrated that the patterns of variation in “vessel density” and “vessel surface area” in WO and MO are driven mainly by thermoclimatic parameters and mean rainfall, respectively, according to the same reaction norms (Terral et al., 2004). From both ecological and functional viewpoints, wood anatomy in *O. europaea* may be considered as a dynamic compromise between (1) water transport efficiency through forming large vessels and (2) safe sap conduction associated both with narrower, numerous, and joined vessels to limit the risks of cavitation and embolism and with structural support (Terral et al., 2004).

At the intrapopulation level, variations in the slope of the linear models and antagonistically in *y*-intercept were significantly explained by aridity (Anderegg et al., 2016). For WO and MO, the greater the intensity of aridity (low value of AI) experienced by populations, the lower the value of slope and the higher the value of *y*-intercept shown. Nevertheless, within comparable bioclimatic and vegetation formation conditions, the slope varied according to (1) the additional water resources provided by an intermittent stream (e.g., “Asni” population) and (2) VC. In the latter case, the observed lower slope may be explained by the degradation of tree and shrub layers (e.g., under the dry subhumid conditions of “Mesmouda” and “Tnin Sidi Yemeni” and within traditional agroecosystems). Both scenarios have been associated with a strong increase in potential evapotranspiration and a severe loss of water resources at a local scale (Terral et al., 2004; Fonti et al., 2009). From a functional viewpoint, a trend of changes in sap conduction strategy and a trade-off between xylem efficiency and safety, modulated by the cambium functioning, is evident (Maherali et al., 2004). Olive trees growing under favorable conditions regarding water resources (dry subhumid, subhumid/humid, and semiarid streamside conditions) differ in their wood anatomy from trees growing under drought stress (semiarid and dry subhumid within traditional agroecosystem; Figure 5).

### Changes in Local Conditions Induce Changes in Anatomical Traits

Compared with the individuals growing in optimal conditions, wild olive trees under lower water resources seem to produce a wood with higher hydraulic conductivity early in branch development. The linear trend of increase in hydraulic conductivity against branch growth appears to be only moderate as a result of the higher risk of drought-induced embolism (Figure 5). These theoretical models are used to identify the risks incurred by the wild olive tree in the event of an increasing frequency in periods of drought, in particular the inability of the cambium to produce large enough vessels to transport sufficient sap to initiate growth and to sustain the high demands of the developing young axes (Choat et al., 2018). Consequently, the inability to produce larger vessels during the development of the growth unit is potentially lethal to the branch of the tree and in consequence to the persistence of the population.

*O. europaea* subspecies (WO and MO in our study area) are both drought tolerant and able to tolerate a large water deficit and high solar radiation, temperatures, and evapotranspiration rates. These characteristics result from a range of morphological, anatomical, and physiological mechanisms. The small size and the relative high density of the stomata contribute to the effective control of stomatal transpiration (Connor, 2005). Moreover, a range of leaf traits of Moroccan populations of wild olive (WO) (e.g., dry matter content, surface area, and stomata density) were found by Kassout et al. (2019) to be strongly related to ecological parameters associated with aridity. Such traits may be considered as the key elements for understanding physiological tolerance and adaptation to drought of Mediterranean plant species (Kassout et al., 2019).

## Conclusion and Perspectives

The complexity of underlying factors behind the responses of hydraulic traits makes it difficult to highlight direct relationships between drought and variation in the anatomical traits of wood. This is particularly true under natural conditions where multiple interactions between ecological parameters operate both within individual trees and between populations. Nonetheless, the established reaction norms illustrating the anatomical plasticity of wild olive within our study illustrate how the functioning of the cambium modulates and controls sap conduction, according to available water resources. Variations in sap conduction strategy and conductivity trends shed light on the plasticity of wood anatomy of *O. europaea* subsp. *europaea* var. *sylvestris* and *O. europaea* subsp. *maroccana*. This plasticity identifies adaptive, qualitative, and quantitative changes in the capacity of the tree for sap conduction in a heterogeneous and geographically changing environment.

The wild olive subspecies studied here may be used to model responses in Mediterranean vegetation to climate change, particularly the ongoing aridification. Clearly, olive populations located at the Southern limit of their distribution area, impacted by drought stress and human disturbances and whose trend of increase in vessel tend toward the horizontal, are highly threatened. Their individuals will be unable to provide a sufficient volume of the sap necessary for growth and development. Such novel results are potentially key to understanding how the tree utilizes water resources. They can provide new insights into mechanisms allowing resistance and survival in trees under conditions of limited water availability.

## Data Availability Statement

The original contributions presented in the study are included in the article/Supplementary Material, further inquiries can be directed to the corresponding author/s.

## Author Contributions

J-FT and MA conceived the study. JK, J-FT, and MA designed the experiments and methodology. All the coauthors collected the material. JK, MA, SI, HB, BL, JR, and J-FT collected the data. LP managed the geographical data. JK and J-FT analyzed the eco-anatomical data and led the writing of the manuscript. All authors contributed critically to the drafts and gave final approval for publication.

## Conflict of Interest

The authors declare that the research was conducted in the absence of any commercial or financial relationships that could be construed as a potential conflict of interest.

## Supplementary Material

Supplementary Figure 1 General presentation of the studied populations. GC, geographical coordinates; Alt, altitude; PC, plant community; AI, aridity index (UNEP, 1997); VC, vegetation coverage.

Supplementary Figure 2 Cumulative average curves allowing us to estimate the number of measurements required for a reliable estimate of an anatomical trait.

Supplementary Figure 3 Linear regression models of vessel surface area (SVS, μm^2^) in relation to the branch diameter (mm) and diagram of standardized residuals for each population [**(A)** population 1–6, **(B)** population 7–12, and **(C)** population 13, for population details see Table 1).

Supplementary Figure 4 Correlation network between vessel density (DVS), vessel surface area (SVS), and number of vessel joined in radial files (NVS).

Supplementary Table 1 Normality tests (Shapiro-Wilk) of the eco-anatomical features measured during two sessions carried out by two different operators.

Supplementary Table 2 Normality tests (Shapiro-Wilk) of the eco-anatomical features measured during two sessions carried out by the same operators.

Supplementary Table 3 Results of mean comparison tests (*t-*test for data sets following a normal distribution, Mann-Whitney in the contrary case) of two sessions carried out by two different operators.

Supplementary Table 4 Results of mean comparison tests (*t-*test for data sets following a normal distribution, Mann-Whitney in the contrary case) of two sessions carried out by the same operators.

Supplementary Table 5 Eco-anatomical data and diameter (mean values) of wood samples for each population alphabetically listed.

Supplementary Table 6 Variance decomposition of vessel surface area (SVS) across the studied levels.

Supplementary Table 7 Pair-wise comparisons provided of differences in Standardized Major Axis regression slopes for the regression between vessel surface area (SVS) and branch diameter (BD) of the studied samples grouped according to their climatic context.

Supplementary Table 8 Pair-wise comparisons provided of differences in Standardized Major Axis regression slopes and elevation for the regression between vessel surface area (SVS) and branch diameter for the studied samples grouped into seven groups according to their climatic and vegetation characteristics.

## References

[B1] Albert C. H. Thuiller W. Yoccoz N. G. Soudant A. Boucher F. Saccone P.. (2010). Intraspecific functional variability: extent, structure and sources of variation. J. Ecol. 98, 604–613. 10.1111/j.1365-2745.2010.01651.x

[B2] Allen C. D. Macalady A. K. Chenchouni H. Bachelet D. McDowell N. Vennetier M.. (2010). A global overview of drought and heat-induced tree mortality reveals emerging climate change risks for forests. For. Ecol. Manag. 259, 660–684. 10.1016/j.foreco.2009.09.001

[B3] Anderegg W. R Klein T. Bartlett M. Sack L. Pellegrini A. F. Choat B.. (2016). Meta-analysis reveals that hydraulic traits explain cross-species patterns of drought-induced tree mortality across the globe. Proc. Natl. Acad. Sci. U.S.A. 113, 5024–5029. 10.1073/pnas.1525678113 27091965 PMC4983847

[B4] Anderegg W. R. J. Meinzer F. C. (2015). Wood anatomy and plant hydraulics in a changing climate, in Functional and Ecological Xylem Anatomy, ed Hacke U. (Cham:Springer), 235–253.

[B5] Anderegg W. R. L. Berry J. A. Smith D. D. Sperry J. S. Anderegg L. D. L. Field C. B. (2012). The roles of hydraulic and carbon stress in a widespread climate-induced forest die-off. Proc. Natl. Acad. Sci. U.S.A. 109, 233–237. 10.1073/pnas.1107891109 22167807 PMC3252909

[B6] Aumeeruddy-Thomas Y. Hmimsa Y. Ater M. Khadari B. (2014). Beyond the divide betweeen wild and domesticated: spaciality, domesticity and practices pertaining to fig (*Ficus carica* L.) and olive (*Olea europaea* L.) agroecosystems among Jbala communities in Northern Morocco, in Plants and People. Choices and Diversity Through Time, ed Chevalier A. Marinova E. Peña-Chocarro L. (Oxford: Oxbow Books (EARTH)), 191–197.

[B7] Benabid A. Fennane M. (1994). Connaissances sur la végétation du Maroc: phytogéographie, phytosociologie et séries de végétation. Lazaroa 14, 21–97.

[B8] Braun-Blanquet J. (1964). Pflanzensoziologie. Grundzüge der Vegetationskunde. Vienna: Springer-Verlag. 10.1007/978-3-7091-8110-2

[B9] Brodribb T. J. Powers J. Cochard H. Choat B. (2020). Hanging by a thread? Forests and drought. Science 368, 261–266. 10.1126/science.aat7631 32299945

[B10] Carlquist S. (1988). Comparative Wood Anatomy - Systemic, Ecological, and Evolutionary Aspects of Dicotyledon Wood. Berlin: Springer Verlag.

[B11] Choat B. Brodribb T. J. Brodersen C. R. Duursma R. A. López R. Medlyn B. E. (2018). Triggers of tree mortality under drought. Nature 558, 531–539. 10.1038/s41586-018-0240-x 29950621

[B12] Choat B. Jansen S. Brodribb T. J. Cochard H. Delzon S. Bhaskar R.. (2012). Global convergence in the vulnerability of forests to drought. Nature 491, 752–755. 10.1038/nature11688 23172141

[B13] Connor D. (2005). Adaptation of olive (*Olea europaea* L.) to water-limited environments. Aust. J. Agric. Res. 56, 1181–1189. 10.1071/AR05169

[B14] Cramer W. Guiot J. Fader M. Garrabou J. Gattuso J.-P. Iglesias A.. (2018). Climate change and interconnected risks to sustainable development in the Mediterranean. Nat. Clim. Change 8, 972–980. 10.1038/s41558-018-70299-2

[B15] Eckstein D. Frisse E. (1979). Environmental influences on the vessel size of beech and oak. IAWA Bull. 2-3, 36–37.

[B16] Ennajeh M. Vadel A. M. Cochard H. Khemir H. (2010). Comparative impacts of water stress on the leaf anatomy of a drought-resistant and a drought-sensitive olive cultivar. J. Hortic. Sci. Biotechnol. 85, 289–294. 10.1080/14620316.2010.11512670

[B17] Fick S. Hijmans R. (2017). WorldClim2: new 1-km spatial resolution climate surfaces for global land areas. Int. J. Climatol. 37, 4302–4315. 10.1002/joc.5086

[B18] Figueiral I. Terral J.-F. (2002). Late Quaternary refugia of Mediterranean taxa in the Portuguese Estremadura: charcoal based palaeovegetation and climatic reconstruction. Q. Sci. Rev. 21, 549–558. 10.1016/S0277-3791(01)00022-1

[B19] Fonti P. von Arx G. García-González I. Eilmann B. Sass-Klaassen U. Gärtner H.. (2009). Studying global change through investigation of the plastic responses of xylem anatomy in tree rings. New Phytol. 185, 42–53. 10.1111/j.1469-8137.2009.03030.x 19780986

[B20] Gianguzzi L. Bazan G. (2019). The Olea europaea L. var. sylvestris (Mill.) Lehr. forests in the Mediterranean area. Plant Sociol. 56, 3–4. 10.7338/pls2019562/01

[B21] Guerfel M. Beis A. Zotos T. Boujnah D. Zarrouk M. Patakas A. (2009). Differences in abscisic acid concentration in roots and leaves of two young olive (*Olea europaea* L.) cultivars in response to water deficit. Acta Physiol. Plant. 31, 825–831. 10.1007/s11738-009-0298-z

[B22] Hampe A. Petit R. J. (2005). Conserving biodiversity under climate change: the rear edge matters. Ecol. Lett. 8:461–467. 10.1111/j.1461-0248.2005.00739.x 21352449

[B23] Kassout J. Barbara H. Ivorra S. Terral J.-F. Ater M. (2016). Etude préliminaire de la variation de caractères anatomiques du bois d'une forme spontanée et de sept variétés traditionnelles d'olivier (Olea europaeaL.) de la région Nord du Maroc (Chefchaouen et Ouazzane),, in L'oléiculture au Maroc de la préhistoire à nos jours: pratiques, diversité, adaptation, usages, commerce et politiques, eds Ater M. Essalouh L. Ilbert H. Moukhli A. Khadari B. (Montpellier: CIHEAM, Options Méditerranéennes: Série A. Séminaires Méditerranéens; n. 118), 181–189.

[B24] Kassout J. Terral J.-F. Hodgson J. Ater M. (2019). Trait-based plant ecology a flawed tool in climate studies? The leaf traits of wild olive that pattern with climate are not those routinely measured. PLoS ONE 14:e0219908. 10.1371/journal.pone.0219908 31314789 PMC6636763

[B25] Lamy J. B. Bouffier L. Burlett R. Plomion C. Cochard H. Delzon S. (2011). Uniform selection as a primary force reducing population genetic differentiation of cavitation resistance across a species range. PLoS ONE 6:e23476. 10.1371/journal.pone.0023476 21858137 PMC3155568

[B26] Leal S. Sousa V. Pereira H. (2007). Radial variation of vessel size and distribution in cork oak wood (*Quercus suber* L.). Wood Sci. Technol. 41, 339–350. 10.1007/s00226-006-0112-7

[B27] Limier B. Ivorra S. Bouby L. Figueiral I. Chabal L. Cabanis M.. (2018). Documenting the history of the grapevine and viticulture: a quantitative eco-anatomical perspective applied to modern and archaeological charcoal. J. Arch. Sci. 100, 45–61. 10.1016/j.jas.2018.10.001

[B28] Lo Gullo M. A. Salleo S. (1988). Different strategies of drought-resistance in three Mediterranean sclerophyllous trees growing in the same environmental conditions. New Phytol. 108, 267–276. 10.1111/j.1469-8137.1988.tb04162.x 33873932

[B29] Maherali H. Pockman W. T. Jackson R. B. (2004). Adaptive variation in the vulnerability of woody plants to xylem cavitation. Ecology 85, 2184–2199. 10.1890/02-0538

[B30] Médail F. Quézel P. Besnard G. Khadari B. (2001). Systematics, ecology and phylogeographic significance of *Olea europaea* L. ssp. maroccana (Greuter and Burdet) P. Vargas et al., a relictual olive tree in south-west Morocco. Bot. J. Linn. Soci. 137, 249–266. 10.1006/bojl.2001.0477

[B31] Paradis E. Claude J. Strimmer K. (2004). APE: analyses of phylogenetics and evolution in R Language. Bioinformatics 20, 289–290. 10.1093/bioinformatics/btg412 14734327

[B32] Parmesan C. Yohe G. (2003). A globally coherent fingerprint of climate change impacts across natural systems. Nature 421, 37–42. 10.1038/nature01286 12511946

[B33] Perez-Martin A. Michelazzo C. Torres-Ruiz J. M. Flexas J. Fernández J. E. Sebastiani L.. (2014). Regulation of photosynthesis and stomatal and mesophyll conductance under water stress and recovery in olive trees: correlation with gene expression of carbonic anhydrase and aquaporins. J. Exp. Bot. 65, 3143–3156. 10.1093/jxb/eru160 24799563 PMC4071832

[B34] Pielke R. A. Pitman A. Niyogi D. Mahmood R. McAlpine C. Hossain F.. (2011). Land use/land cover changes and climate: modeling analysis and observational evidence. Rev. Clim. Change 2, 828–850. 10.1002/wcc.144

[B35] Pinheiro J. Bates D. Debroy S. Sarkar D. R Core Team (2019). nlme: Linear and Nonlinear Mixed Effects Models. R package version 3.1-141. Available online at: https://CRAN.R-project.org/package=nlme

[B36] Quero J. L. Sterck F. J. Martínez-Vilalta J. Villar R. (2011). Water-use strategies of six co-existing Mediterranean woody species during a summer drought. Oecologia 166, 45–57. 10.1007/s00442-011-1922-3 21290148

[B37] R Development Core Team (2015). R: A Language and Environment for Statistical Computing. Vienna: R Foundation for Statistical Computing.

[B38] Ros J. Terral J.-F. Ruas M.-P. Ivorra S. Limier B. Paradis L.. (2021). Understanding anatomical plasticity of Argan wood features at local geographical scale in ecological and archaeobotanical perspectives. Sci. Rep. 11:10830. 10.1038/s41598-021-90286-4 34031505 PMC8144426

[B39] Rosner S. Klein A. Müller U. Karlsson B. (2007). Hydraulic and mechanical properties of young Norway spruce clones related to growth and wood structure. Tree Physiol. 27, 1165–1178. 10.1093/treephys/27.8.1165 17472942 PMC3197722

[B40] Steppe K. Sterck F. Deslauries A. (2015). Diel growth dynamics in tree stems: linking anatomy and ecophysiology. Trends Plant Sci. 20, 335–343. 10.1016/j.tplants.2015.03.015 25911419

[B41] Terral J.-F. (2000). Exploitation and management of the olive tree during prehistoric times in Mediterranean France and Spain. J. Arch. Sci. 27, 127–133. 10.1006/jasc.1999.0444

[B42] Terral J.-F. Arnold-Simard G. (1996). Beginnings of olive cultivation in Eastern Spain in relation to Holocene bioclimatic changes. Q. Res. 46, 176–185. 10.1006/qres.1996.0057

[B43] Terral J.-F. Badal E. Heinz C. Roiron P. Thiébault S. Figueiral I. (2004). A hydraulic conductivity model points to post-Neogene survival of the Mediterranean Olive in riparian habitat. Ecology 85, 3158–3165. 10.1890/03-308113

[B44] Terral J.-F. Durand A. (2006). Bio-archaeological evidence of olive tree (*Olea europaea* L.) irrigation during the Middle Ages in Southern France and North Eastern Spain. J. Archaeol. Sci. 33, 718–724. 10.1016/j.jas.2005.10.004

[B45] Trifilò F. Lo Gullo M. A. Nardini A. Pernice F. Salleo S. (2007). Rootstock effects on xylem conduit dimensions and vulnerability to cavitation of *Olea europaea* L. Trees Struct. Funct. 21, 549–556. 10.1007/s00468-007-0148-9

[B46] UNEP (1997). World Atlas of Desertification, 2nd Edn. London: Arnold.

[B47] Volaire F. (2018). A unified framework of plant adaptive strategies to drought: crossing scales and disciplines. Glob. Change Biol. 24, 2929–2938. 10.1111/gcb.14062 29350812

[B48] Voulgaridis E. (1990). Wood cell morphology characteristics of some oak species and mediterranean shrubs. Euro. J. W. W. Prod. 48, 261–267. 10.1007/BF02626512

[B49] Warton D. I. Duursma R. A. Falster D. S. Taskinen S. (2012). SMATR 3 – an R package for estimation and inference about allometric lines. Methods Ecol. Evol. 3, 257–259. 10.1111/j.2041-210X.2011.00153.x

[B50] West A. G. Dawson T. E. February E. C. Midgley G. F. Bond W. J. Aston T. L. (2012). Diverse functional responses to drought in a Mediterranean-type shrubland in South Africa. New Phytol. 195, 396–407. 10.1111/j.1469-8137.2012.04170.x 22594652

[B51] Woodcock DW. (1989). Climate sensitivity of wood-anatomical features in a ring-porous oak (*Quercus macrocarpa*). Rev. Can. Rech. For. 19, 639–644. 10.1139/x89-100

[B52] Zomer R. J. Bossio D. A. Trabucco A. Yuanjie L. Gupta D. C. Singh V. P. (2007). Trees and Water: Smallholder Agroforestry on Irrigated Lands in Northern India. IWMI Research Report 122.2007. 10.1016/j.agee.2008.01.014

