# Supplementary figures and images for: Resisting Aridification: Adaptation of Sap Conduction Performance in Moroccan Wild Olive Subspecies Distributed Over an Aridity Gradient
Source: Front Plant Sci. 2021 Jul 2;12:663721. doi: 10.3389/fpls.2021.663721 (PMC8283533; doi:10.3389/fpls.2021.663721)

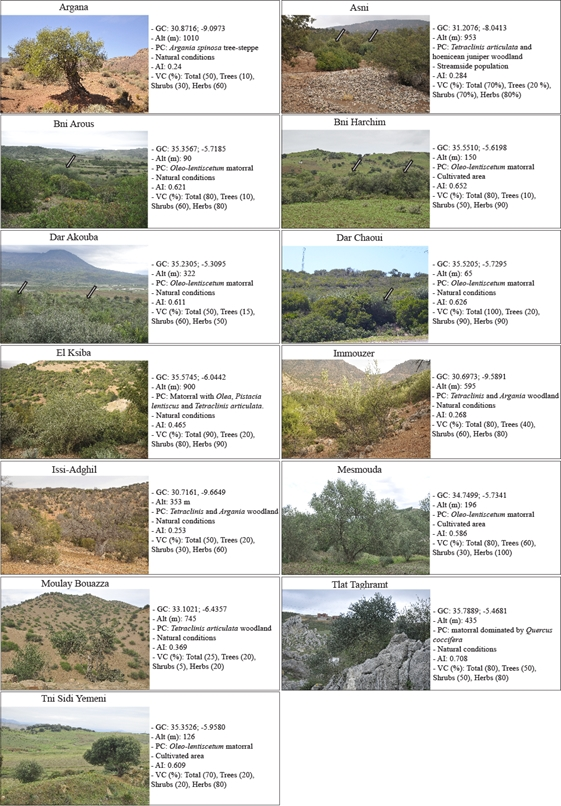

Supplement: Supplementary Figure 1 — General presentation of the studied populations. GC, geographical coordinates; Alt, altitude; PC, plant community; AI, aridity index (UNEP, 1997); VC, vegetation coverage. [file Image_1.TIFF]

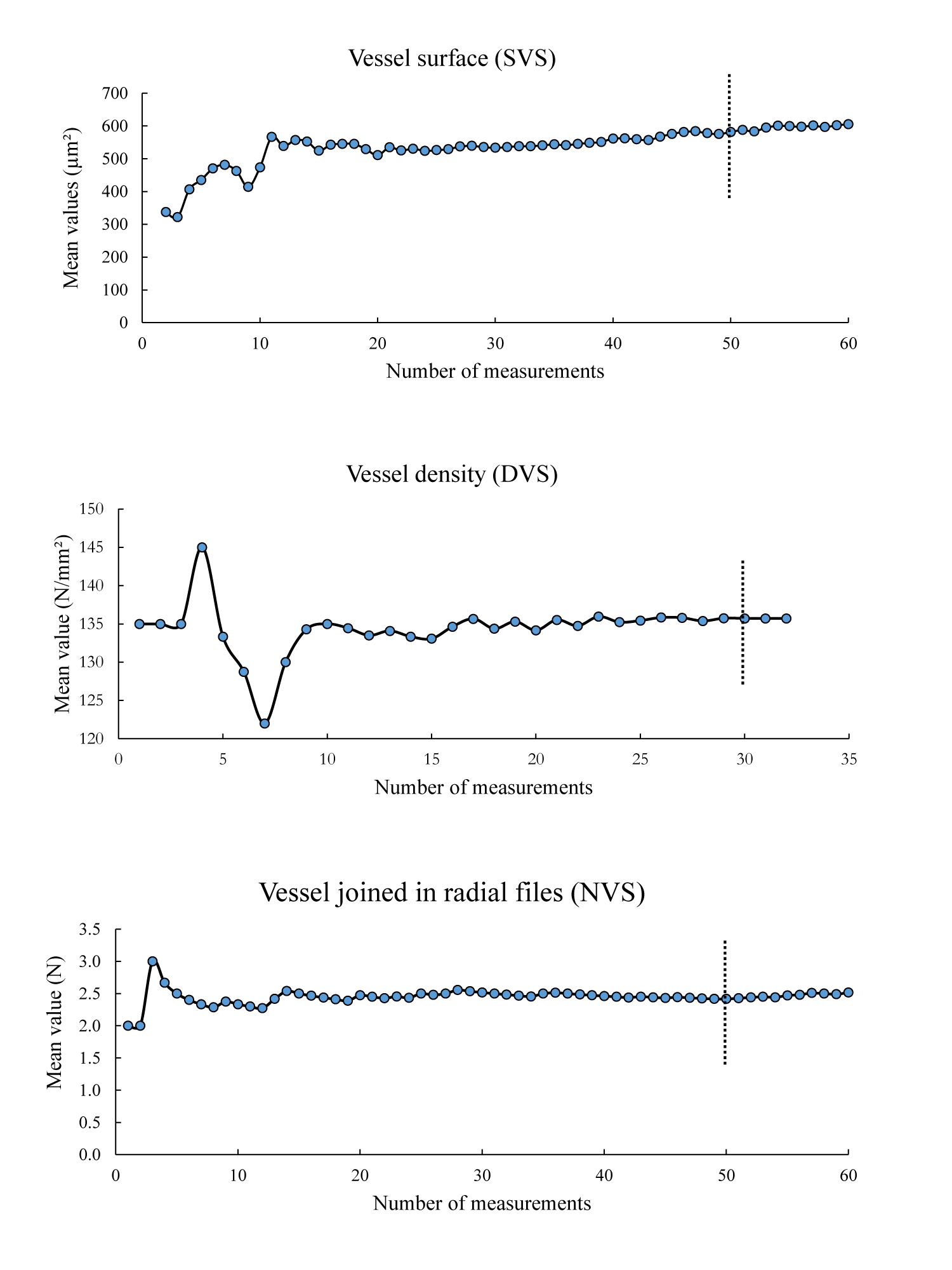

Supplement: Supplementary Figure 2 — Cumulative average curves allowing us to estimate the number of measurements required for a reliable estimate of an anatomical trait. [file Image_2.TIF]

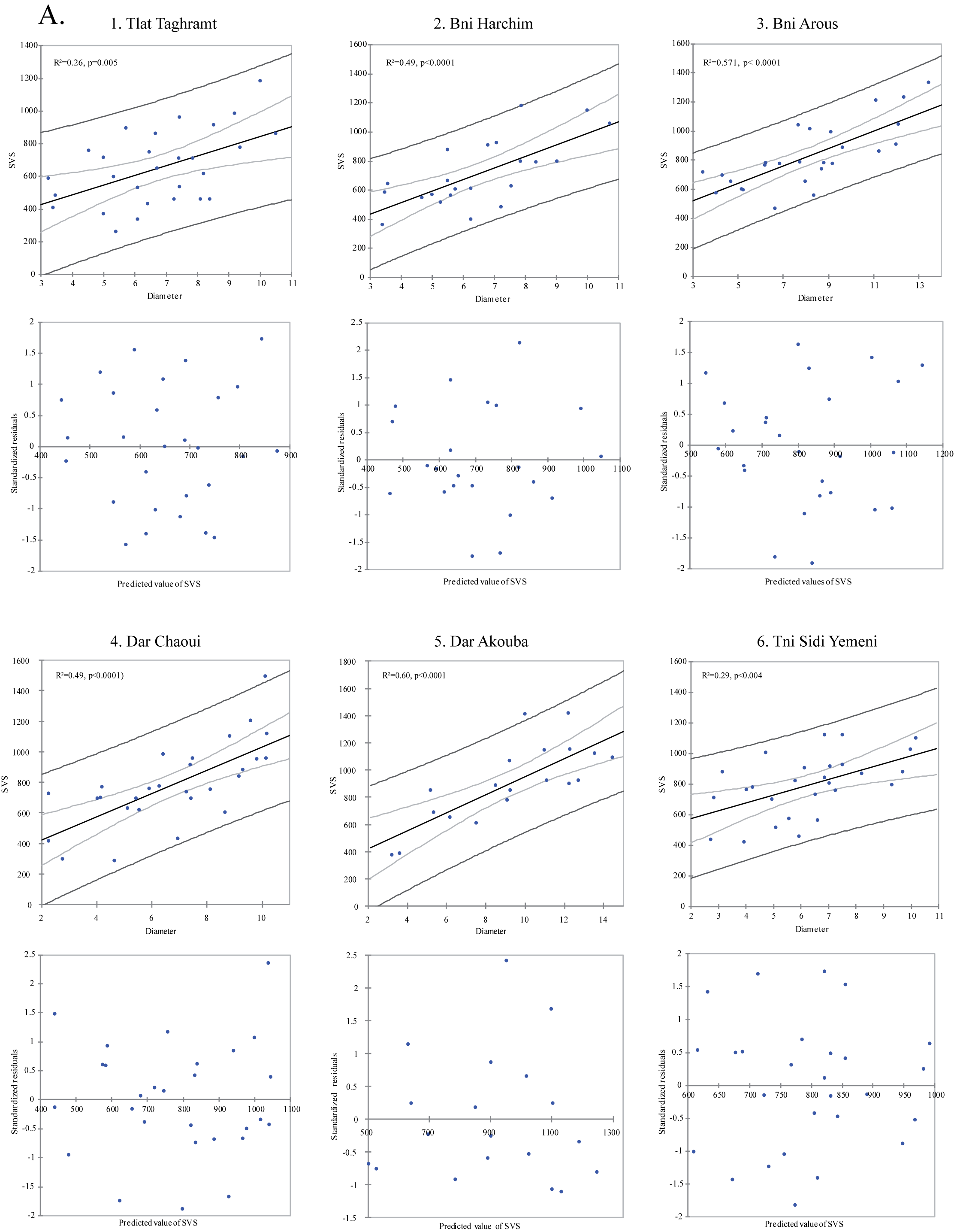

Supplement: Supplementary file 3 [file Image_3.TIF]

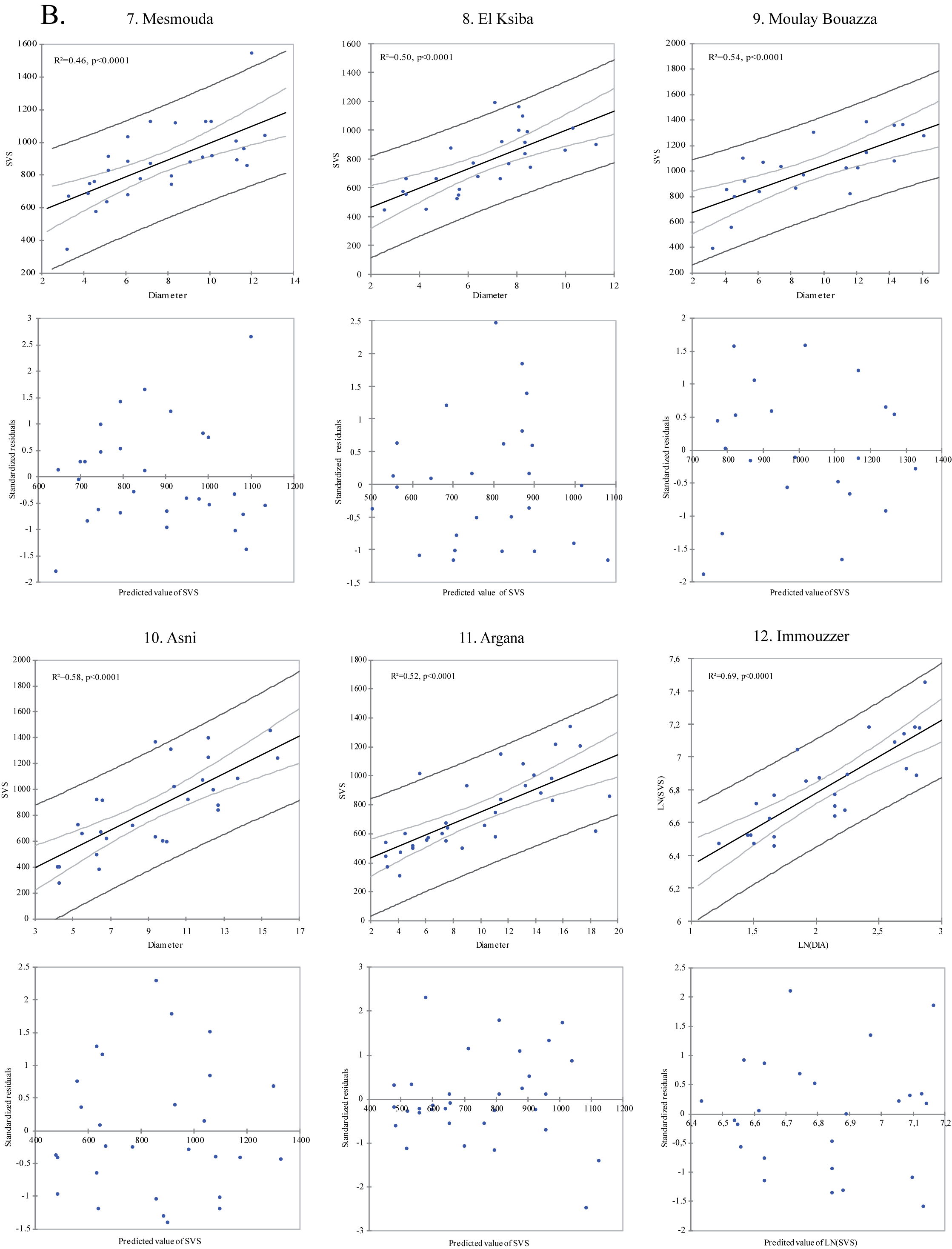

Supplement: Supplementary file 4 [file Image_4.TIF]

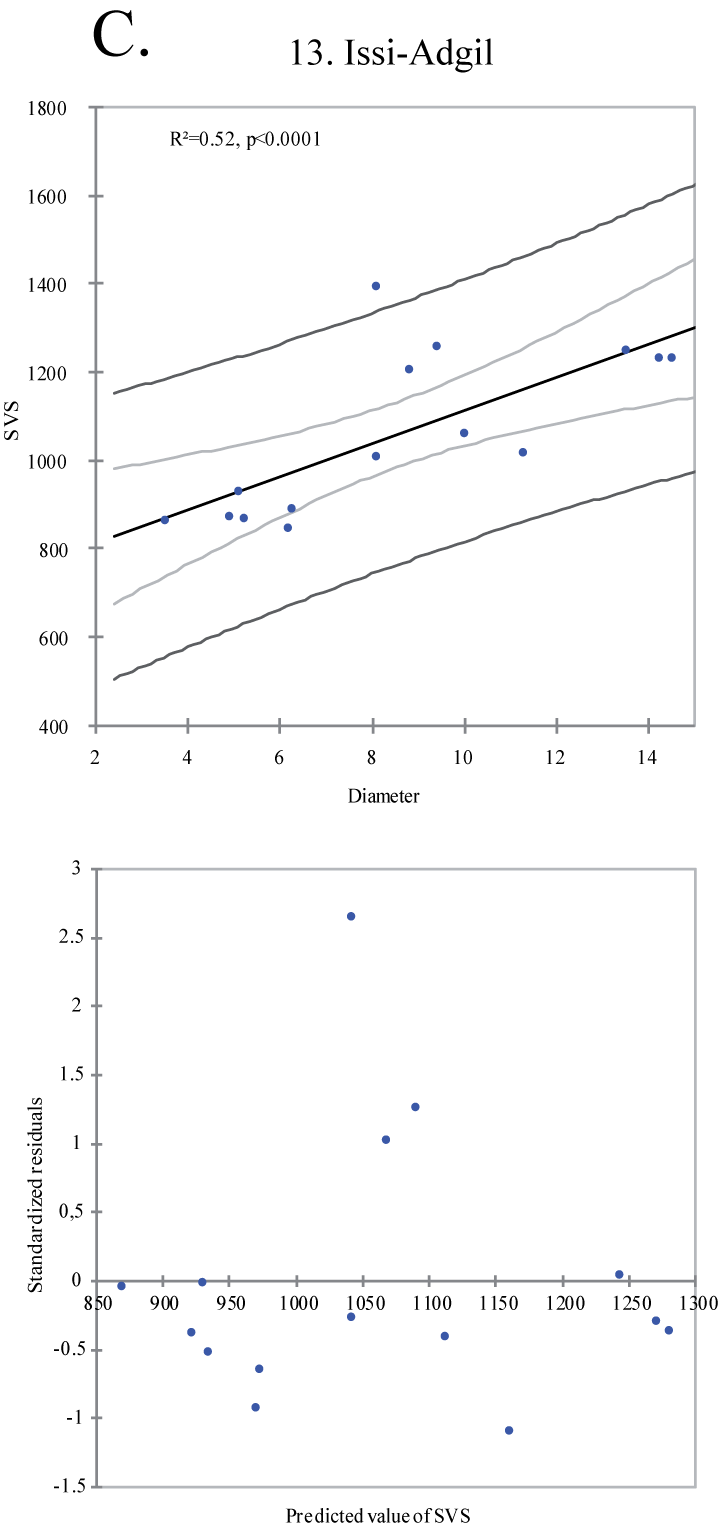

Supplement: Supplementary Figure 3 — Linear regression models of vessel surface area (SVS, μm2) in relation to the branch diameter (mm) and diagram of standardized residuals for each population [(A) population 1–6, (B) population 7–12, and (C) population 13, for population details see Table 1). [file Image_5.TIF]

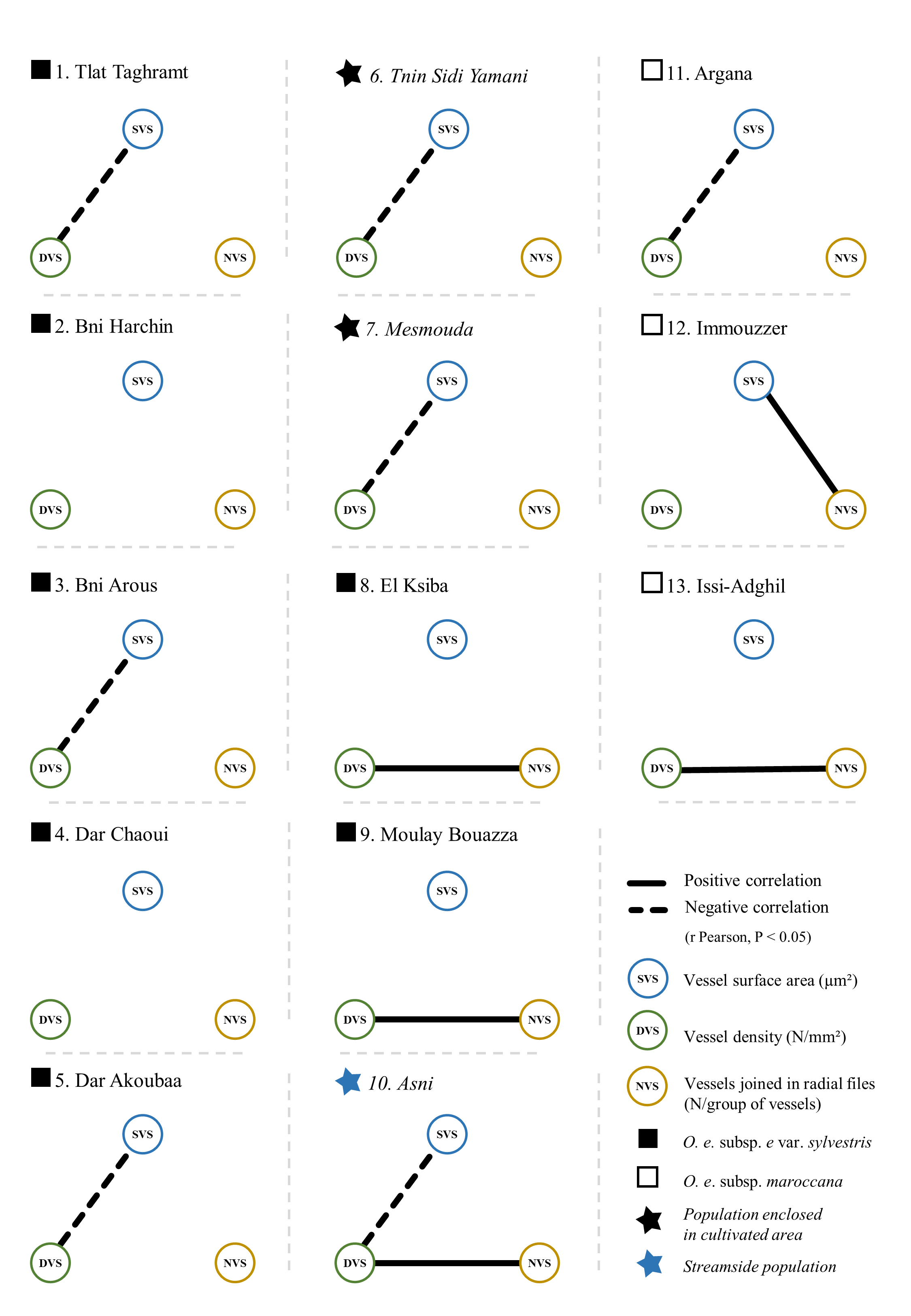

Supplement: Supplementary Figure 4 — Correlation network between vessel density (DVS), vessel surface area (SVS), and number of vessel joined in radial files (NVS). [file Image_6.TIF]
