# Supplementary material for: Resisting Aridification: Adaptation of Sap Conduction Performance in Moroccan Wild Olive Subspecies Distributed Over an Aridity Gradient
Source: Front Plant Sci. 2021 Jul 2;12:663721. doi: 10.3389/fpls.2021.663721 (PMC8283533; doi:10.3389/fpls.2021.663721)
Supplement: Supplementary Table 1 — Normality tests (Shapiro-Wilk) of the eco-anatomical features measured during two sessions carried out by two different operators. [file Data_Sheet_1.docx]

**Supplementary Tables**

**Table S1.** Normality tests (Shapiro-Wilk) of the eco-anatomical features measured during two sessions carried out by two different operators.

| Measurment sessions | Characters | W | p-value | Normality |
| --- | --- | --- | --- | --- |
| Operator 1 (BR) | SVS | 0.956 | 0.064 | yes |
|  | DVS | 0.952 | 0.194 | yes |
| Operator 2 (JA) | SVS | 0.958 | 0.077 | yes |
|  | DVS | 0.966 | 0.458 | yes |

**Table S2.** Normality tests (Shapiro-Wilk) of the eco-anatomical features measured during two sessions carried out by the same operators.

| Measurement sessions | Characters | W | p-value | Normality |
| --- | --- | --- | --- | --- |
| Session 1 (A) | SVS | 0.960 | 0.091 | yes |
|  | DVS | 0.981 | 0.860 | yes |
| Session 2 (B) | SVS | 0.978 | 0.496 | yes |
|  | DVS | 0.961 | 0.329 | yes |

**Table S3.** Results of mean comparison tests (*t-*test for data sets following a normal distribution, Mann-Whitney in the contrary case) of two sessions carried out by two different operators.

| *Student t-test* | |  |  |  |  |
| --- | --- | --- | --- | --- | --- |
| Characters | t (observed values) | \|t\| (Critical value) | df | p-value | Significance |
| SVS | -0.845 | 1.984 | 98 | 0.400 | ns |
| DVS | 0.820 | 2.002 | 58 | 0.416 | ns |
| *Mann-Whitney* | | | | | |
| Characters | U test | | p-value | | Significance |
| NVS | 1299.0 | | 0.652 | | ns |

ns, non significant

**Table S4.** Results of mean comparison tests (*t-*test for data sets following a normal distribution, Mann-Whitney in the contrary case) of two sessions carried out by the same operators.

| *Student t-test* |  |  |  |  |  |
| --- | --- | --- | --- | --- | --- |
| Characters | t (observed values) | \|t\| (Critical value) | df | p-value | Significance |
| SVS | 0.191 | 1.984 | 98 | 0.849 | ns |
| DVS | -0.822 | 2.002 | 58 | 0.414 | ns |
| *Mann-Whitney* | | | | | |
| Characters | U test | | p-value | | Significance |
| NVS | 1114.0 | | 0.265 | | ns |

ns, non significant

**Table S5.** Eco-anatomical data and diameter (mean values) of wood samples for each population alphabetically listed.

| Population | ID ISEM-sample | DVS (N/mm²) | SVS (µm²) | NVS (N/group) | Diameter (mm) | Conditions |
| --- | --- | --- | --- | --- | --- | --- |
| Argana | ARGA4-1 | 213.57 | 1148.66 | 2.68 | 11.51 | natural |
| Argana | ARGA4-2 | 143.04 | 551.88 | 2.12 | 6.10 | natural |
| Argana | ARGA4-3 | 297.19 | 879.13 | 2.82 | 14.40 | natural |
| Argana | ARGA5-1 | 120.12 | 1336.87 | 2.54 | 16.55 | natural |
| Argana | ARGA5-2 | 253.69 | 927.56 | 2.12 | 13.30 | natural |
| Argana | ARGA5-3 | 227.98 | 832.49 | 2.22 | 11.50 | natural |
| Argana | ARGA6-1 | 300.48 | 857.64 | 3.24 | 19.43 | natural |
| Argana | ARGA6-2 | 411.90 | 366.66 | 2.46 | 3.20 | natural |
| Argana | ARGA6-3 | 370.60 | 672.70 | 2.34 | 7.50 | natural |
| Argana | ARGA6-4 | 314.76 | 744.30 | 2.16 | 11.10 | natural |
| Argana | ARGA7-1 | 184.17 | 1078.85 | 2.56 | 13.10 | natural |
| Argana | ARGA7-2 | 203.06 | 536.86 | 2.20 | 3.10 | natural |
| Argana | ARGA7-3 | 109.05 | 617.38 | 2.18 | 18.40 | natural |
| Argana | ARGA8-1 | 251.07 | 1002.37 | 4.08 | 13.90 | natural |
| Argana | ARGA8-2 | 237.28 | 596.48 | 2.32 | 4.50 | natural |
| Argana | ARGA8-3 | 171.07 | 928.01 | 2.38 | 9.00 | natural |
| Argana | ARGA9-1 | 136.19 | 826.92 | 2.76 | 15.23 | natural |
| Argana | ARGA9-2 | 286.61 | 443.30 | 2.44 | 3.10 | natural |
| Argana | ARGA9-3 | 296.67 | 549.37 | 2.28 | 7.50 | natural |
| Argana | ARGA9-4 | 157.98 | 978.93 | 2.50 | 15.20 | natural |
| Argana | ARGA10-1 | 140.12 | 1217.51 | 2.66 | 15.48 | natural |
| Argana | ARGA10-2 | 130.65 | 1012.81 | 2.14 | 5.60 | natural |
| Argana | ARGA10-3 | 80.24 | 497.92 | 2.16 | 8.70 | natural |
| Argana | ARGA11-1 | 123.21 | 1201.96 | 2.56 | 17.30 | natural |
| Argana | ARGA11-2 | 341.54 | 517.10 | 2.24 | 5.10 | natural |
| Argana | ARGA11-3 | 217.98 | 599.43 | 2.48 | 7.20 | natural |
| Argana | ARGA12-1 | 309.98 | 638.99 | 2.36 | 7.60 | natural |
| Argana | ARGA12-2 | 313.74 | 471.57 | 2.34 | 4.20 | natural |
| Argana | ARGA13-2 | 191.79 | 656.99 | 2.96 | 10.30 | natural |
| Argana | ARGA13-3 | 235.09 | 498.87 | 3.06 | 5.10 | natural |
| Argana | ARGA14-1 | 197.14 | 307.55 | 2.37 | 4.10 | natural |
| Argana | ARGA14-2 | 206.58 | 572.68 | 2.32 | 6.20 | natural |
| Argana | ARGA14-3 | 159.88 | 576.99 | 2.28 | 11.10 | natural |
| Argana | ARGA15-1 | 120.05 | 459.64 | 2.21 | 6.30 | streamside |
| Argana | ARGA15-2 | 136.07 | 791.06 | 2.34 | 13.40 | streamside |
| Argana | ARGA16-1 | 236.73 | 508.66 | 2.52 | 4.40 | streamside |
| Argana | ARGA16-2 | 149.40 | 919.62 | 2.66 | 9.50 | streamside |
| Argana | ARGA1-1 | 211.79 | 1180.33 | 2.88 | 15.27 | streamside |
| Argana | ARGA1-2 | 113.78 | 1213.70 | 2.08 | 5.11 | streamside |
| Argana | ARGA1-3 | 86.19 | 1284.52 | 2.28 | 9.50 | streamside |
| Argana | ARGA2-1 | 227.98 | 1033.21 | 3.04 | 10.30 | streamside |
| Argana | ARGA2-2 | 128.81 | 917.24 | 2.28 | 7.20 | streamside |
| Argana | ARGA2-3 | 86.31 | 1016.25 | 2.20 | 13.60 | streamside |
| Argana | ARGA2-4 | 121.67 | 1243.99 | 2.32 | 10.30 | streamside |
| Asni | ASNI1-1 | 194.64 | 1072.20 | 2.74 | 11.90 | streamside |
| Asni | ASNI1-2 | 296.43 | 617.32 | 3.02 | 6.80 | streamside |
| Asni | ASNI2-1 | 139.64 | 1308.24 | 2.46 | 10.20 | streamside |
| Asni | ASNI2-2 | 323.35 | 667.15 | 2.94 | 6.50 | streamside |
| Asni | ASNI3-1 | 128.21 | 1394.42 | 2.74 | 12.20 | streamside |
| Asni | ASNI3-2 | 130.71 | 917.45 | 2.24 | 6.30 | streamside |
| Asni | ASNI3-3 | 91.90 | 1362.50 | 2.34 | 9.40 | streamside |
| Asni | ASNI4-1 | 157.74 | 1247.24 | 2.38 | 12.20 | streamside |
| Asni | ASNI4-2 | 162.82 | 652.37 | 2.24 | 5.50 | streamside |
| Asni | ASNI4-3 | 120.83 | 910.58 | 2.08 | 6.60 | streamside |
| Asni | ASNI5-1 | 169.52 | 1452.38 | 2.56 | 15.50 | streamside |
| Asni | ASNI5-2 | 233.26 | 725.71 | 2.18 | 5.30 | streamside |
| Asni | ASNI5-3 | 107.86 | 919.17 | 2.16 | 11.10 | streamside |
| Asni | ASNI6-1 | 169.40 | 993.87 | 3.04 | 12.48 | streamside |
| Asni | ASNI6-2 | 140.91 | 489.31 | 2.26 | 6.30 | streamside |
| Asni | ASNI6-3 | 92.98 | 591.87 | 2.20 | 10.00 | streamside |
| Asni | ASNI7-1 | 263.57 | 835.12 | 3.16 | 12.70 | streamside |
| Asni | ASNI7-2 | 309.74 | 274.52 | 2.56 | 4.30 | streamside |
| Asni | ASNI7-3 | 143.39 | 376.81 | 2.46 | 6.40 | streamside |
| Asni | ASNI7-4 | 187.38 | 627.99 | 2.74 | 9.40 | streamside |
| Asni | ASNI8-1 | 189.17 | 1084.02 | 3.06 | 13.75 | streamside |
| Asni | ASNI8-2 | 223.51 | 398.41 | 2.40 | 4.20 | streamside |
| Asni | ASNI8-3 | 170.50 | 599.58 | 2.68 | 9.80 | streamside |
| Asni | ASNI9-1 | 222.98 | 873.60 | 3.16 | 12.70 | streamside |
| Asni | ASNI9-2 | 176.19 | 396.55 | 2.18 | 4.30 | streamside |
| Asni | ASNI9-3 | 272.86 | 715.38 | 2.26 | 8.20 | streamside |
| Asni | ASNI10-1 | 151.79 | 1236.05 | 2.72 | 15.89 | streamside |
| Asni | ASNI10-2 | 152.83 | 654.10 | 2.34 | 5.50 | streamside |
| Asni | ASNI10-3 | 209.76 | 1017.85 | 2.18 | 10.40 | streamside |
| Bni Arous | BNA1-1 | 106.07 | 1231.04 | 2.12 | 12.34 | natural |
| Bni Arous | BNA1-2 | 107.14 | 1040.67 | 2.64 | 7.69 | natural |
| Bni Arous | BNA1-3 | 219.29 | 717.59 | 2.10 | 3.44 | natural |
| Bni Arous | BNA2-1 | 122.89 | 994.67 | 2.18 | 9.13 | natural |
| Bni Arous | BNA2-2 | 129.40 | 468.72 | 2.78 | 6.64 | natural |
| Bni Arous | BNA2-3 | 256.75 | 600.24 | 2.20 | 5.18 | natural |
| Bni Arous | BNA3-1 | 162.50 | 781.29 | 2.36 | 8.82 | natural |
| Bni Arous | BNA3-2 | 149.05 | 772.96 | 2.72 | 6.85 | natural |
| Bni Arous | BNA3-3 | 207.31 | 592.76 | 2.34 | 5.24 | natural |
| Bni Arous | BNA5-1 | 80.95 | 1045.22 | 2.10 | 12.10 | natural |
| Bni Arous | BNA5-2 | 80.95 | 653.19 | 2.10 | 7.98 | natural |
| Bni Arous | BNA5-3 | 187.95 | 697.13 | 2.26 | 4.29 | natural |
| Bni Arous | BNA6-1 | 183.10 | 776.82 | 2.16 | 9.20 | natural |
| Bni Arous | BNA6-2 | 172.38 | 654.53 | 3.22 | 4.70 | natural |
| Bni Arous | BNA7-1 | 131.67 | 1212.93 | 2.54 | 11.10 | natural |
| Bni Arous | BNA7-3 | 128.93 | 1012.95 | 2.22 | 8.19 | natural |
| Bni Arous | BNA8-1 | 77.38 | 1333.49 | 2.20 | 13.43 | natural |
| Bni Arous | BNA8-2 | 113.21 | 787.59 | 2.50 | 7.75 | natural |
| Bni Arous | BNA9-1 | 94.64 | 858.20 | 2.14 | 11.26 | natural |
| Bni Arous | BNA9-2 | 104.52 | 778.32 | 2.56 | 6.23 | natural |
| Bni Arous | BNA9-3 | 96.07 | 739.90 | 2.22 | 8.72 | natural |
| Bni Arous | BNA10-1 | 98.66 | 908.01 | 2.18 | 12.02 | natural |
| Bni Arous | BNA10-2 | 124.29 | 889.04 | 2.92 | 9.63 | natural |
| Bni Arous | BNA10-3 | 130.95 | 557.58 | 2.36 | 8.36 | natural |
| Bni Arous | BNA7-2 | 192.31 | 762.81 | 2.50 | 6.18 | natural |
| Bni Arous | BNA9-4 | 143.57 | 570.65 | 2.26 | 4.02 | natural |
| Bni Arous | BNA4-1 | 97.26 | 1501.99 | 2.12 | 9.81 | streamside |
| Bni Arous | BNA4-2 | 118.88 | 981.36 | 2.02 | 8.60 | streamside |
| Bni Arous | BNA4-3 | 87.62 | 952.68 | 2.81 | 4.13 | streamside |
| Bni Harchin | BNH1-1 | 107.59 | 793.51 | 2.06 | 8.36 | natural |
| Bni Harchin | BNH1-3 | 130.60 | 400.19 | 2.60 | 6.25 | natural |
| Bni Harchin | BNH1-2 | 217.26 | 363.32 | 2.32 | 3.40 | natural |
| Bni Harchin | BNH2-1 | 190.91 | 875.66 | 2.56 | 5.50 | natural |
| Bni Harchin | BNH2-2 | 138.69 | 641.76 | 3.40 | 3.58 | natural |
| Bni Harchin | BNH3-1 | 175.27 | 911.14 | 2.54 | 6.79 | natural |
| Bni Harchin | BNH3-2 | 242.98 | 561.13 | 3.12 | 5.61 | natural |
| Bni Harchin | BNH4-1 | 218.41 | 628.67 | 2.26 | 7.56 | natural |
| Bni Harchin | BNH4-3 | 260.71 | 605.36 | 2.44 | 5.76 | natural |
| Bni Harchin | BNH4-2 | 130.71 | 587.44 | 2.58 | 3.47 | natural |
| Bni Harchin | BNH5-1 | 162.50 | 548.95 | 2.28 | 4.67 | natural |
| Bni Harchin | BNH5-2 | 114.64 | 612.63 | 2.40 | 6.25 | natural |
| Bni Harchin | BNH6-1 | 149.40 | 1057.72 | 2.24 | 10.72 | natural |
| Bni Harchin | BNH6-2 | 156.90 | 923.82 | 2.96 | 7.07 | natural |
| Bni Harchin | BNH6-3 | 174.35 | 515.26 | 2.31 | 5.27 | natural |
| Bni Harchin | BNH7-1 | 120.48 | 796.26 | 2.72 | 9.03 | natural |
| Bni Harchin | BNH7-2 | 99.18 | 485.85 | 2.06 | 7.22 | natural |
| Bni Harchin | BNH8-1 | 181.43 | 1178.31 | 2.72 | 7.88 | natural |
| Bni Harchin | BNH8-2 | 134.78 | 567.29 | 2.32 | 5.01 | natural |
| Bni Harchin | BNH9-1 | 121.67 | 1146.48 | 2.86 | 10.01 | natural |
| Bni Harchin | BNH9-2 | 104.52 | 798.03 | 2.24 | 7.86 | natural |
| Bni Harchin | BNH9-3 | 141.23 | 662.40 | 2.31 | 5.50 | natural |
| Bni Harchin | BNH10-1 | 117.14 | 1375.82 | 2.22 | 9.08 | natural |
| Bni Harchin | BNH10-2 | 102.50 | 1225.42 | 2.12 | 7.65 | natural |
| Dar Akouba | DAR1-1 | 132.02 | 851.86 | 2.68 | 9.25 | natural |
| Dar Akouba | DAR1-2 | 104.05 | 1142.88 | 2.10 | 11.00 | natural |
| Dar Akouba | DAR1-3 | 208.39 | 611.24 | 2.26 | 7.51 | natural |
| Dar Akouba | DAR2-1 | 107.00 | 885.58 | 2.42 | 8.50 | natural |
| Dar Akouba | DAR2-2 | 96.55 | 1417.36 | 2.38 | 12.22 | natural |
| Dar Akouba | DAR3-1 | 89.17 | 1408.76 | 2.18 | 10.00 | natural |
| Dar Akouba | DAR3-2 | 91.31 | 1121.75 | 2.18 | 13.56 | natural |
| Dar Akouba | DAR3-3 | 111.43 | 849.94 | 2.40 | 5.20 | natural |
| Dar Akouba | DAR4-1 | 99.67 | 923.06 | 2.32 | 11.10 | natural |
| Dar Akouba | DAR4-2 | 171.67 | 897.40 | 2.18 | 12.23 | natural |
| Dar Akouba | DAR5-1 | 103.00 | 778.54 | 2.18 | 9.10 | natural |
| Dar Akouba | DAR5-1 | 80.24 | 1149.29 | 2.10 | 12.30 | natural |
| Dar Akouba | DAR5-2 | 148.85 | 686.86 | 2.10 | 5.34 | natural |
| Dar Akouba | DAR6-3 | 114.17 | 652.51 | 2.18 | 6.17 | natural |
| Dar Akouba | DAR6-4 | 234.52 | 385.54 | 2.12 | 3.64 | natural |
| Dar Akouba | DAR7-1 | 150.60 | 922.90 | 2.96 | 12.71 | natural |
| Dar Akouba | DAR7-2 | 180.22 | 372.75 | 2.28 | 3.24 | natural |
| Dar Akouba | DAR7-3 | 148.69 | 1064.87 | 2.30 | 9.23 | natural |
| Dar Akouba | DAR8-1 | 73.93 | 1093.97 | 2.38 | 14.44 | natural |
| Dar Chaoui | DAC1-1 | 88.45 | 1398.07 | 2.28 | 10.17 | natural |
| Dar Chaoui | DAC1-2 | 106.79 | 981.97 | 2.28 | 6.42 | natural |
| Dar Chaoui | DAC1-3 | 124.33 | 697.78 | 2.10 | 4.15 | natural |
| Dar Chaoui | DAC1-4 | 133.57 | 414.77 | 2.08 | 2.27 | natural |
| Dar Chaoui | DAC2-1 | 124.40 | 1119.15 | 2.12 | 10.19 | natural |
| Dar Chaoui | DAC2-2 | 170.71 | 736.28 | 3.40 | 7.27 | natural |
| Dar Chaoui | DAC2-3 | 142.65 | 691.72 | 2.18 | 4.04 | natural |
| Dar Chaoui | DAC3-1 | 111.79 | 1493.29 | 2.22 | 10.12 | natural |
| Dar Chaoui | DAC3-2 | 103.81 | 754.15 | 2.26 | 8.12 | natural |
| Dar Chaoui | DAC3-3 | 98.81 | 432.90 | 2.08 | 6.95 | natural |
| Dar Chaoui | DAC3-4 | 93.45 | 285.32 | 2.20 | 4.65 | natural |
| Dar Chaoui | DAC4-1 | 118.93 | 880.38 | 2.52 | 9.32 | natural |
| Dar Chaoui | DAC4-2 | 86.25 | 603.12 | 2.08 | 8.66 | natural |
| Dar Chaoui | DAC5-1 | 132.86 | 950.90 | 3.06 | 9.83 | natural |
| Dar Chaoui | DAC5-2 | 187.20 | 626.91 | 2.14 | 5.12 | natural |
| Dar Chaoui | DAC6-1 | 198.14 | 836.57 | 2.20 | 9.17 | natural |
| Dar Chaoui | DAC6-2 | 169.76 | 616.70 | 2.62 | 5.56 | natural |
| Dar Chaoui | DAC7-1 | 165.48 | 913.40 | 2.54 | 7.41 | natural |
| Dar Chaoui | DAC7-2 | 156.55 | 759.46 | 2.08 | 5.92 | natural |
| Dar Chaoui | DAC7-3 | 101.07 | 295.40 | 2.22 | 2.78 | natural |
| Dar Chaoui | DAC8-1 | 130.24 | 1103.77 | 2.30 | 8.84 | natural |
| Dar Chaoui | DAC8-2 | 110.89 | 957.60 | 2.04 | 7.49 | natural |
| Dar Chaoui | DAC8-3 | 161.73 | 693.30 | 2.16 | 5.43 | natural |
| Dar Chaoui | DAC9-1 | 157.98 | 691.98 | 3.60 | 7.44 | natural |
| Dar Chaoui | DAC9-2 | 65.58 | 956.52 | 2.22 | 10.15 | natural |
| Dar Chaoui | DAC10-1 | 102.83 | 1204.01 | 2.06 | 9.59 | natural |
| Dar Chaoui | DAC10-2 | 143.75 | 727.21 | 2.20 | 2.26 | natural |
| Dar Chaoui | DAC10-3 | 101.19 | 773.71 | 2.06 | 6.27 | natural |
| Dar Chaoui | DAC10-4 | 137.26 | 768.36 | 2.80 | 4.20 | natural |
| El Ksiba | EK1-1 | 124.83 | 997.44 | 2.52 | 8.10 | natural |
| El Ksiba | EK1-3 | 127.81 | 586.87 | 2.44 | 5.65 | natural |
| El Ksiba | EK1-2 | 70.36 | 1013.50 | 2.22 | 10.30 | natural |
| El Ksiba | EK2-1 | 141.33 | 520.27 | 2.56 | 5.55 | natural |
| El Ksiba | EK2-2 | 97.86 | 986.67 | 2.32 | 8.45 | natural |
| El Ksiba | EK3-1 | 121.33 | 914.01 | 2.34 | 8.36 | natural |
| El Ksiba | EK3-2 | 113.27 | 658.42 | 2.10 | 4.71 | natural |
| El Ksiba | EK4-1 | 149.33 | 766.49 | 2.98 | 7.69 | natural |
| El Ksiba | EK4-2 | 95.54 | 855.94 | 2.22 | 10.00 | natural |
| El Ksiba | EK4-3 | 124.68 | 554.07 | 2.38 | 3.45 | natural |
| El Ksiba | EK5-1 | 139.17 | 900.24 | 2.78 | 11.26 | natural |
| El Ksiba | EK5-2 | 107.14 | 742.21 | 2.42 | 8.56 | natural |
| El Ksiba | EK6-1 | 126.33 | 1187.25 | 2.32 | 7.11 | natural |
| El Ksiba | EK6-2 | 118.51 | 658.53 | 2.06 | 3.45 | natural |
| El Ksiba | EK7-1 | 119.50 | 1097.34 | 2.40 | 8.26 | natural |
| El Ksiba | EK7-2 | 133.57 | 677.80 | 2.50 | 6.40 | natural |
| El Ksiba | EK7-3 | 82.62 | 919.82 | 2.54 | 7.40 | natural |
| El Ksiba | EK8-1 | 143.17 | 1157.44 | 2.40 | 8.10 | natural |
| El Ksiba | EK8-2 | 143.41 | 572.12 | 2.32 | 3.33 | natural |
| El Ksiba | EK8-3 | 112.14 | 661.60 | 2.32 | 7.34 | natural |
| El Ksiba | EK9-1 | 144.00 | 831.56 | 2.50 | 8.35 | natural |
| El Ksiba | EK9-2 | 109.76 | 448.80 | 2.14 | 4.29 | natural |
| El Ksiba | EK10-1 | 127.67 | 770.13 | 2.46 | 6.22 | natural |
| El Ksiba | EK10-2 | 92.86 | 443.74 | 2.16 | 2.56 | natural |
| El Ksiba | EK10-3 | 60.48 | 547.36 | 2.16 | 5.61 | natural |
| El Ksiba | EK11 | 69.40 | 871.59 | 2.06 | 5.30 | natural |
| Immouzer | IMMO1-1 | 142.14 | 1019.94 | 2.74 | 15.20 | natural |
| Immouzer | IMMO1-2 | 171.43 | 635.12 | 2.24 | 5.30 | natural |
| Immouzer | IMMO1-3 | 91.23 | 811.65 | 2.36 | 8.60 | natural |
| Immouzer | IMMO2-1 | 107.14 | 1200.22 | 2.52 | 13.88 | natural |
| Immouzer | IMMO2-2 | 138.49 | 646.10 | 2.40 | 4.50 | natural |
| Immouzer | IMMO2-3 | 79.62 | 963.95 | 2.32 | 7.60 | natural |
| Immouzer | IMMO3-1 | 162.62 | 1308.89 | 2.64 | 11.34 | natural |
| Immouzer | IMMO3-2 | 110.15 | 822.62 | 2.26 | 4.60 | natural |
| Immouzer | IMMO4-1 | 127.26 | 945.14 | 2.28 | 6.83 | natural |
| Immouzer | IMMO4-2 | 121.43 | 751.91 | 2.22 | 5.10 | natural |
| Immouzer | IMMO4-3 | 55.36 | 872.28 | 2.26 | 8.60 | natural |
| Immouzer | IMMO5-1 | 163.21 | 979.80 | 3.24 | 16.52 | natural |
| Immouzer | IMMO5-2 | 104.85 | 673.17 | 2.42 | 5.30 | natural |
| Immouzer | IMMO5-3 | 84.05 | 761.30 | 2.46 | 8.60 | natural |
| Immouzer | IMMO6-1 | 166.79 | 1261.43 | 2.66 | 15.00 | natural |
| Immouzer | IMMO6-2 | 193.37 | 679.77 | 2.06 | 4.30 | natural |
| Immouzer | IMMO6-3 | 136.16 | 981.13 | 2.08 | 9.50 | natural |
| Immouzer | IMMO8-1 | 130.83 | 1301.91 | 2.66 | 16.90 | natural |
| Immouzer | IMMO8-2 | 118.97 | 867.75 | 2.16 | 5.30 | natural |
| Immouzer | IMMO8-3 | 146.07 | 792.18 | 2.26 | 9.30 | natural |
| Immouzer | IMMO9-1 | 103.33 | 1726.41 | 2.54 | 17.70 | natural |
| Immouzer | IMMO9-2 | 112.99 | 645.37 | 2.10 | 3.40 | natural |
| Immouzer | IMMO9-3 | 91.19 | 1144.61 | 2.04 | 6.40 | natural |
| Immouzer | IMMO10-1 | 142.02 | 1314.40 | 2.94 | 16.31 | natural |
| Immouzer | IMMO10-2 | 124.18 | 679.39 | 2.16 | 4.40 | natural |
| Issi-Adghil | ISSI1-1 | 124.00 | 888.15 | 2.30 | 6.25 | natural |
| Issi-Adghil | ISSI1-2 | 104.76 | 1232.03 | 2.30 | 14.50 | natural |
| Issi-Adghil | ISSI2-1 | 106.33 | 1391.45 | 2.46 | 8.10 | natural |
| Issi-Adghil | ISSI2-2 | 210.71 | 928.30 | 2.62 | 5.10 | natural |
| Issi-Adghil | ISSI3-1 | 161.79 | 1249.32 | 2.84 | 13.50 | natural |
| Issi-Adghil | ISSI3-2 | 164.76 | 1058.97 | 2.68 | 10.00 | natural |
| Issi-Adghil | ISSI4-1 | 184.29 | 1231.73 | 2.76 | 14.25 | natural |
| Issi-Adghil | ISSI4-2 | 202.68 | 847.63 | 2.70 | 6.20 | natural |
| Issi-Adghil | ISSI5-1 | 193.10 | 1016.80 | 3.00 | 11.30 | natural |
| Issi-Adghil | Adghi 1 L2 | 105.97 | 871.39 | 2.46 | 4.90 | natural |
| Issi-Adghil | Adghi 1 L6 | 118.33 | 1005.78 | 2.60 | 8.10 | natural |
| Issi-Adghil | Adghi 2 L2 | 111.33 | 864.20 | 2.36 | 3.49 | natural |
| Issi-Adghil | Adghi 2 L5 | 108.10 | 1202.54 | 2.56 | 8.80 | natural |
| Issi-Adghil | Adghi 3 L3 | 91.67 | 865.77 | 2.24 | 5.23 | natural |
| Issi-Adghil | Adghi 3 L5 | 123.57 | 1257.40 | 2.86 | 9.40 | natural |
| Mesmouda | MES1-1 | 91.19 | 915.31 | 2.36 | 10.14 | agroecosytem |
| Mesmouda | MES1-2 | 160.71 | 686.72 | 2.46 | 4.20 | agroecosytem |
| Mesmouda | MES1-3 | 158.04 | 911.98 | 2.32 | 5.20 | agroecosytem |
| Mesmouda | MES2-1 | 227.50 | 893.05 | 3.14 | 11.31 | agroecosytem |
| Mesmouda | MES2-2 | 185.71 | 826.26 | 2.70 | 5.20 | agroecosytem |
| Mesmouda | MES3-1 | 149.64 | 1006.00 | 3.06 | 11.27 | agroecosytem |
| Mesmouda | MES3-2 | 183.33 | 869.09 | 2.64 | 7.20 | agroecosytem |
| Mesmouda | MES3-3 | 238.93 | 747.95 | 2.70 | 4.30 | agroecosytem |
| Mesmouda | MES4-1 | 220.48 | 741.05 | 3.54 | 8.20 | agroecosytem |
| Mesmouda | MES4-2 | 324.55 | 636.27 | 2.86 | 5.10 | agroecosytem |
| Mesmouda | MES4-3 | 331.43 | 576.60 | 2.74 | 4.60 | agroecosytem |
| Mesmouda | MES5-1 | 99.29 | 961.47 | 2.20 | 11.65 | agroecosytem |
| Mesmouda | MES5-2 | 177.62 | 879.78 | 2.62 | 9.10 | agroecosytem |
| Mesmouda | MES5-3 | 192.86 | 882.58 | 2.36 | 6.10 | agroecosytem |
| Mesmouda | MES5-4 | 251.59 | 758.86 | 2.50 | 4.50 | agroecosytem |
| Mesmouda | MES5-5 | 177.62 | 858.90 | 2.50 | 11.80 | agroecosytem |
| Mesmouda | MES6-1 | 141.43 | 1125.15 | 2.94 | 9.83 | agroecosytem |
| Mesmouda | MES6-2 | 258.21 | 678.96 | 3.12 | 6.10 | agroecosytem |
| Mesmouda | MES7-1 | 246.90 | 650.90 | 3.56 | 10.83 | agroecosytem |
| Mesmouda | MES7-2 | 279.22 | 530.35 | 2.78 | 5.90 | agroecosytem |
| Mesmouda | MES8-1 | 233.21 | 1125.37 | 3.36 | 10.10 | agroecosytem |
| Mesmouda | MES8-2 | 492.86 | 670.73 | 2.28 | 3.30 | agroecosytem |
| Mesmouda | MES8-3 | 184.76 | 1127.48 | 2.82 | 7.20 | agroecosytem |
| Mesmouda | MES9-1 | 156.67 | 907.45 | 2.28 | 9.67 | agroecosytem |
| Mesmouda | MES9-2 | 255.00 | 777.85 | 2.58 | 6.70 | agroecosytem |
| Mesmouda | MES9-3 | 289.29 | 343.06 | 2.36 | 3.20 | agroecosytem |
| Mesmouda | MES10-1 | 136.07 | 1544.11 | 3.36 | 12.02 | agroecosytem |
| Mesmouda | MES10-2 | 193.10 | 1032.22 | 2.60 | 6.10 | agroecosytem |
| Mesmouda | MES10-3 | 167.14 | 1119.37 | 2.58 | 8.40 | agroecosytem |
| Mesmouda | MES11-1 | 122.26 | 1042.27 | 2.64 | 12.66 | agroecosytem |
| Mesmouda | MES11-2 | 179.17 | 792.69 | 2.58 | 8.20 | agroecosytem |
| Moulay Bouazza | MBO1-1 | 105.00 | 1077.08 | 2.40 | 14.30 | natural |
| Moulay Bouazza | MBO1-2 | 140.80 | 394.39 | 2.20 | 3.24 | natural |
| Moulay Bouazza | MBO1-3 | 127.26 | 1064.57 | 2.20 | 6.34 | natural |
| Moulay Bouazza | MBO3-1 | 89.76 | 1021.86 | 2.62 | 12.10 | natural |
| Moulay Bouazza | MBO3-2 | 92.62 | 917.57 | 2.30 | 5.21 | natural |
| Moulay Bouazza | MBO5-1 | 113.81 | 865.73 | 2.40 | 8.32 | natural |
| Moulay Bouazza | MBO5-2 | 109.17 | 1360.17 | 2.14 | 14.30 | natural |
| Moulay Bouazza | MBO6-1 | 126.31 | 1143.82 | 2.42 | 12.62 | natural |
| Moulay Bouazza | MBO6-2 | 194.75 | 800.28 | 2.54 | 4.60 | natural |
| Moulay Bouazza | MBO6-3 | 187.36 | 968.99 | 2.76 | 8.80 | natural |
| Moulay Bouazza | MBO7-1 | 90.48 | 1363.94 | 2.58 | 14.83 | natural |
| Moulay Bouazza | MBO7-2 | 198.93 | 851.13 | 2.68 | 4.10 | natural |
| Moulay Bouazza | MBO7-3 | 183.57 | 1301.54 | 2.82 | 9.40 | natural |
| Moulay Bouazza | MBO8-1 | 168.10 | 821.17 | 2.46 | 11.62 | natural |
| Moulay Bouazza | MBO8-2 | 179.29 | 835.28 | 2.64 | 6.10 | natural |
| Moulay Bouazza | MBO9-1 | 112.26 | 1383.17 | 2.36 | 12.63 | natural |
| Moulay Bouazza | MBO9-2 | 225.71 | 1102.18 | 2.74 | 5.10 | natural |
| Moulay Bouazza | MBO9-3 | 190.36 | 1031.16 | 2.60 | 7.40 | natural |
| Moulay Bouazza | MBO10-1 | 125.71 | 1274.58 | 2.50 | 16.09 | natural |
| Moulay Bouazza | MBO10-2 | 237.00 | 557.06 | 2.70 | 4.40 | natural |
| Moulay Bouazza | MBO10-3 | 140.57 | 1023.40 | 2.60 | 11.40 | natural |
| Tlat Taghramt | TLA1-1 | 159.17 | 596.69 | 2.68 | 5.33 | natural |
| Tlat Taghramt | TLA2-1 | 163.83 | 896.44 | 2.54 | 5.71 | natural |
| Tlat Taghramt | TLA2-3 | 156.87 | 716.37 | 2.42 | 5.00 | natural |
| Tlat Taghramt | TLA2-4 | 96.27 | 756.53 | 2.18 | 4.54 | natural |
| Tlat Taghramt | TLA2-2 | 73.45 | 963.62 | 2.30 | 7.43 | natural |
| Tlat Taghramt | TLA3-1 | 160.00 | 860.66 | 2.70 | 6.67 | natural |
| Tlat Taghramt | TLA3-2 | 153.78 | 589.32 | 2.74 | 3.23 | natural |
| Tlat Taghramt | TLA3-3 | 221.94 | 536.23 | 2.94 | 7.44 | natural |
| Tlat Taghramt | TLA4-1 | 120.17 | 1184.81 | 2.34 | 10.00 | natural |
| Tlat Taghramt | TLA4-2 | 155.36 | 649.62 | 2.44 | 6.71 | natural |
| Tlat Taghramt | TLA5-1 | 88.00 | 912.04 | 2.18 | 8.52 | natural |
| Tlat Taghramt | TLA5-3 | 182.89 | 710.18 | 2.32 | 7.40 | natural |
| Tlat Taghramt | TLA5-2 | 213.10 | 372.45 | 2.34 | 5.00 | natural |
| Tlat Taghramt | TLA6-1 | 112.50 | 775.01 | 2.32 | 9.37 | natural |
| Tlat Taghramt | TLA6-3 | 130.00 | 482.73 | 2.14 | 3.45 | natural |
| Tlat Taghramt | TLA6-2 | 264.29 | 337.80 | 2.43 | 6.10 | natural |
| Tlat Taghramt | TLA7-1 | 101.00 | 860.06 | 2.46 | 10.51 | natural |
| Tlat Taghramt | TLA7-2 | 147.32 | 614.56 | 2.60 | 8.20 | natural |
| Tlat Taghramt | TLA8-1 | 136.67 | 750.96 | 2.34 | 6.46 | natural |
| Tlat Taghramt | TLA8-2 | 98.21 | 458.44 | 2.05 | 8.10 | natural |
| Tlat Taghramt | TLA9-1 | 133.81 | 459.09 | 3.40 | 7.25 | natural |
| Tlat Taghramt | TLA9-2 | 230.22 | 406.63 | 2.28 | 3.40 | natural |
| Tlat Taghramt | TLA9-3 | 90.11 | 260.73 | 2.18 | 5.40 | natural |
| Tlat Taghramt | TLA10-1 | 127.86 | 984.22 | 2.58 | 9.19 | natural |
| Tlat Taghramt | TLA10-2 | 180.36 | 462.07 | 2.24 | 8.40 | natural |
| Tlat Taghramt | TLA10-3 | 95.71 | 431.67 | 2.12 | 6.40 | natural |
| Tlat Taghramt | TLA11-1 | 186.90 | 712.25 | 3.18 | 7.84 | natural |
| Tlat Taghramt | TLA11-2 | 224.29 | 532.66 | 2.58 | 6.10 | natural |
| Tni Sidi Yemeni | TNI1-1 | 149.17 | 803.40 | 2.64 | 7.04 | agroecosytem |
| Tni Sidi Yemeni | TNI1-2 | 148.32 | 762.70 | 2.22 | 4.03 | agroecosytem |
| Tni Sidi Yemeni | TNI2-2 | 77.68 | 564.65 | 2.00 | 6.62 | agroecosytem |
| Tni Sidi Yemeni | TNI3-1 | 150.60 | 865.85 | 2.94 | 8.20 | agroecosytem |
| Tni Sidi Yemeni | TNI3-2 | 185.99 | 573.81 | 2.44 | 5.57 | agroecosytem |
| Tni Sidi Yemeni | TNI3-3 | 147.77 | 729.94 | 2.48 | 6.52 | agroecosytem |
| Tni Sidi Yemeni | TNI5-1 | 107.32 | 1101.74 | 2.18 | 10.18 | agroecosytem |
| Tni Sidi Yemeni | TNI5-2 | 165.71 | 925.96 | 2.84 | 7.50 | agroecosytem |
| Tni Sidi Yemeni | TNI5-3 | 131.51 | 697.25 | 2.16 | 4.95 | agroecosytem |
| Tni Sidi Yemeni | TNI5-4 | 216.67 | 423.12 | 2.64 | 3.93 | agroecosytem |
| Tni Sidi Yemeni | TNI5-5 | 94.40 | 916.49 | 2.22 | 7.05 | agroecosytem |
| Tni Sidi Yemeni | TNI6-1 | 65.24 | 1122.44 | 2.28 | 6.86 | agroecosytem |
| Tni Sidi Yemeni | TNI6-2 | 152.44 | 1007.49 | 2.42 | 4.74 | agroecosytem |
| Tni Sidi Yemeni | TNI6-2 | 152.98 | 434.39 | 2.28 | 2.72 | agroecosytem |
| Tni Sidi Yemeni | TNI7-1 | 212.30 | 840.78 | 2.22 | 6.86 | agroecosytem |
| Tni Sidi Yemeni | TNI7-2 | 122.14 | 876.25 | 2.56 | 9.70 | agroecosytem |
| Tni Sidi Yemeni | TNI7-3 | 151.65 | 820.67 | 2.14 | 5.80 | agroecosytem |
| Tni Sidi Yemeni | TNI7-4 | 131.90 | 777.93 | 2.92 | 4.26 | agroecosytem |
| Tni Sidi Yemeni | TNI9-1 | 112.74 | 759.31 | 2.22 | 7.26 | agroecosytem |
| Tni Sidi Yemeni | TNI9-2 | 215.18 | 458.99 | 2.32 | 5.93 | agroecosytem |
| Tni Sidi Yemeni | TNI10-1 | 120.48 | 1024.67 | 2.38 | 9.98 | agroecosytem |
| Tni Sidi Yemeni | TNI10-2 | 137.38 | 792.99 | 2.74 | 9.31 | agroecosytem |
| Tni Sidi Yemeni | TNI10-3 | 138.27 | 517.62 | 2.12 | 5.10 | agroecosytem |
| Tni Sidi Yemeni | TNI11-1 | 83.21 | 1120.75 | 2.28 | 7.50 | agroecosytem |
| Tni Sidi Yemeni | TNI11-2 | 131.63 | 905.31 | 2.20 | 6.14 | agroecosytem |
| Tni Sidi Yemeni | TNI11-3 | 71.94 | 877.99 | 2.17 | 3.15 | agroecosytem |
| Tni Sidi Yemeni | TNI11-4 | 157.50 | 709.90 | 2.60 | 2.84 | agroecosytem |
| Tni Sidi Yemeni | TNI4-1 | 202.68 | 399.98 | 2.12 | 6.98 | agroecosytem |
| Tni Sidi Yemeni | TNI4-2 | 138.21 | 775.39 | 2.52 | 9.33 | agroecosytem |
| Tni Sidi Yemeni | TNI8-1 | 82.94 | 1254.85 | 2.12 | 10.47 | agroecosytem |
| Tni Sidi Yemeni | TNI8-2 | 78.93 | 1190.49 | 2.70 | 4.41 | agroecosytem |

**Table S6.** Variance decomposition of vessel surface area (SVS) across the studied levels.

| Level | % of variance |
| --- | --- |
| Sample | 32.54 |
| Population | 0.0005 |
| Aridity | 2.44 |
| Aridity + Vegetation cover (VC) | 0.003 |
| Residual | 65.01 |

**Table S7.** Pair-wise comparisons provided of differences in Standardized Major Axis regression slopes for the regression between vessel surface area (SVS) and branch diameter (BD) of the studied samples grouped according to their climatic context.

|  | Subhumid/humid (Ca1) | Dry subhumid (Ca2) | Semi-arid (Ca3) |
| --- | --- | --- | --- |
| Subhumid/humid  (Ca1) | - | **0.014** | **0.0001** |
| Dry subhumid  (Ca2) |  | - | 0.397 |
| Semi-arid  (Ca3) |  |  | - |

Bold values are significant at P<0.05.

**Table S8.** Pair-wise comparisons provided of differences in Standardized Major Axis regression slopes and elevation for the regression between vessel surface area (SVS) and branch diameter for the studied samples grouped into seven groups according to their climatic and vegetation characteristics.

|  | Cavc1 | Cavc2 | Cavc3 | Cavc4 | Cavc5 | Cavc6 | Cavc7 |
| --- | --- | --- | --- | --- | --- | --- | --- |
| Cavc1 | - | 1.000 | 0.971 | 0.391 | 0.547 | **0.001** | 0.204 |
| Cavc2 |  | - | 0.987 | 0.423 | 0.591 | **0.001** | 0.213 |
| Cavc3 |  |  | - | 1.000 | 1.000 | 0.146 | 1.000 |
| Cavc4 |  |  |  | - | 1.000 | 0.136 | 1.000 |
| Cavc5 |  |  |  |  | - | 0.397 | 1.000 |
| Cavc6 |  |  |  |  |  | - | 0.361 |
| Cavc7 |  |  |  |  |  |  | - |

Bold values are significant at P<0.05. Cavc classes’ abbreviations are described in Materials and Methods section.
